# Type I IFN Triggers RIG-I/TLR3/NLRP3-dependent Inflammasome Activation in Influenza A Virus Infected Cells

**DOI:** 10.1371/journal.ppat.1003256

**Published:** 2013-04-11

**Authors:** Julien Pothlichet, Isabelle Meunier, Beckley K. Davis, Jenny P-Y. Ting, Emil Skamene, Veronika von Messling, Silvia M. Vidal

**Affiliations:** 1 Department of Human Genetics, McGill University, Montreal, Quebec, Canada; 2 McGill Centre for the Study of Host Resistance, McGill University, Montreal, Quebec, Canada; 3 Institut Pasteur, Centre François Jacob, Paris, France; 4 INRS-Institut Armand-Frappier, Laval, Quebec, Canada; 5 Department of Microbiology and Immunology School of Medicine, University of North Carolina at Chapel Hill, Chapel Hill, North Carolina, United States of America; 6 Lineberger Comprehensive Cancer Center, University of North Carolina at Chapel Hill, Chapel Hill, North Carolina, United States of America; Johns Hopkins University - Bloomberg School of Public Health, United States of America

## Abstract

Influenza A virus (IAV) triggers a contagious and potentially lethal respiratory disease. A protective IL-1β response is mediated by innate receptors in macrophages and lung epithelial cells. NLRP3 is crucial in macrophages; however, which sensors elicit IL-1β secretion in lung epithelial cells remains undetermined. Here, we describe for the first time the relative roles of the host innate receptors RIG-I (DDX58), TLR3, and NLRP3 in the IL-1β response to IAV in primary lung epithelial cells. To activate IL-1β secretion, these cells employ partially redundant recognition mechanisms that differ from those described in macrophages. RIG-I had the strongest effect through a MAVS/TRIM25/Riplet–dependent type I IFN signaling pathway upstream of TLR3 and NLRP3. Notably, RIG-I also activated the inflammasome through interaction with caspase 1 and ASC in primary lung epithelial cells. Thus, NS1, an influenza virulence factor that inhibits the RIG-I/type I IFN pathway, strongly modulated the IL-1β response in lung epithelial cells and in ferrets. The NS1 protein derived from a highly pathogenic strain resulted in increased interaction with RIG-I and inhibited type I IFN and IL-1β responses compared to the least pathogenic virus strains. These findings demonstrate that in IAV-infected lung epithelial cells RIG-I activates the inflammasome both directly and through a type I IFN positive feedback loop.

## Author Summary

Although epithelial cells lining our respiratory tract are both the primary targets of influenza A virus (IAV) infection and major players in the outcome of this infection, we still have an imperfect understanding of their response to the virus. Such knowledge is important to design proper anti-influenza strategies. Here, we discovered that epithelial cells sense and respond to IAV infection quite differently than macrophages. While the pathogen recognition receptor (PRR) NLRP3 is crucial in macrophages, we show that IAV infection of primary respiratory epithelial cells triggers IL-1β secretion, which depends not only on NLRP3 but also on two other PRRs, RIG-I and TLR3. In the IL-1β production pathway, RIG-I occupies the most upstream position; it does so directly, through the formation of a RIG-I/ASC inflammasome, and indirectly, through the up-regulation of these three PRRs via a type I IFN positive feedback loop. Further, the increased virulence of IAV in a ferret infection model implicated NS1, a virus protein that targets RIG-I, decreasing both type I IFN and IL-1β secretion in epithelial cells. These results improve our understanding of the tissue-specificity of RIG-I antiviral mechanisms involving type I IFN and IL-1β, and suggest the use of RIG-I agonists in anti-viral therapy or as vaccine adjuvants.

## Introduction

Influenza A virus (IAV) is the etiological agent of a contagious acute respiratory disease. Its seasonal and, more rarely, pandemic strains – such as the deadly 1918 H1N1 virus, are responsible for severe illness and considerable mortality worldwide [1], [2]. The outcome of IAV infections is largely determined by complex interactions between the virus and the innate immune system; therefore, a detailed understanding of the molecular mechanisms involved in innate immune recognition and response can provide a valuable framework for therapeutic development. A protective interleukin 1β (IL-1β) response primarily mediated by macrophages and lung epithelial cells is a key component of the innate immune response to influenza infection [3]. At least four pattern recognition receptors (PRR) that sense IAV infection may be involved: toll-like receptor 3 (TLR3); TLR7; retinoic acid-inducible gene-I (RIG-I, also known as DDX58); and NOD-like receptor family, pyrin domain containing 3 (NLRP3). Lung epithelial cells that line the respiratory tract are the primary targets of influenza infection [4]–[9]. However, the mechanisms of IL-1β secretion in lung epithelial cells remain unresolved. In macrophages and dendritic cells, genomic IAV RNA is detected by TLR7, leading to the stimulation of interferon-α (IFN-α) production [10] and the synthesis of the immature (“pro”) form of IL-1β) [11]. This upregulation of biologically inactive pro-IL-1β is the first signal needed to produce a mature, bioactive form of IL-1β via the formation of an NLRP3 inflammasome. To this end, NLRP3, in complex with the adaptor protein ASC (also known as PYCARD), first induces proteolytic activation of caspase 1. In turn, activated caspase 1 cleaves pro-IL-1β and promotes secretion of mature IL-1β through a nonconventional pathway [11]. Several mechanisms have been proposed to explain NLRP3 inflammasome activation in IAV-infected macrophages, including lysosomal maturation, release of reactive oxygen species [3], and perturbation of ionic concentrations through IAV-encoded M2 ion channels expressed in the *trans* Golgi network [11]. In macrophages, type I IFNs have also been shown to negatively regulate IL-1β production through poorly understood mechanisms [12]. In lung epithelial cells, both TLR3 and RIG-I play a critical role in IAV pathology, and against this infection [4], [13], [14]. TLR3 primarily elicits a pro-inflammatory response upon binding to double-stranded RNA species produced during IAV infection [4]. By contrast, RIG-I recognizes cytosolic single-stranded RNA genomes [15], [16]. By interacting with the mitochondrial adaptor MAVS/IPS-1/cardif/VISA, it elicits both pro-inflammatory and antiviral responses through the transcription factors NF-κB and IRF-3, respectively [4]. Further underscoring their relevance in the host response [17], RIG-I responses are tightly regulated, either positively by TRIM25 [18] and Riplet [19], or negatively by the suppressor of cytokine signaling (SOCS)1 and SOCS3 [5].

On the virus side, the nonstructural protein 1 (NS1) is the main IAV IFN antagonist [2], [18], [20]–[23]. NS1 interacts with RIG-I and its co-activator TRIM25, impairing the activation of the transcription factors that drive IFN-β expression [18], [21]. In addition, in macrophages, NS1 also inhibits caspase 1 activation and IL-1β production [24]. To clarify the host–virus interactions that shape the IL-1β response in human lung epithelial cells, we first examined the relative roles of host innate receptors RIG-I, TLR3, and NLRP3 in the IL-1β response in primary cells. We then analyzed the impact of IAV NS1 on this response in association with virulence in ferrets. We provide evidence that IL-1β secretion is controlled by parallel pathways involving RIG-I/TLR3/NLRP3-dependent inflammasome activation, with RIG-I at the most upstream position. Furthermore, we show that type I IFNs are required for inflammasome activation and that these cytokines mediate RIG-I–dependent regulation of TLR3 and NLRP3 expression. We also demonstrate that RIG-I directly activates the inflammasome by binding to ASC and caspase 1 in primary lung epithelial cells. In support of a role for RIG-I-dependent type I IFN signaling in lung epithelial cells, we show that NS1 modulates IL-1β secretion. Indeed, recombinant viruses carrying NS1 from the highly pathogenic 1918 strain inhibited IL-1β secretion, as they induced a decrease in type I IFN signaling and RIG-I protein expression; this could result from an increased interaction between 1918 NS1 and RIG-I, as opposed to NS1 from other strains. Furthermore, 1918 NS1–dependent virulence correlated with inhibition of both type I IFN and IL-1β expression in IAV-infected ferrets. Altogether, our findings demonstrate that RIG-I is pivotal to the activation of the IL-1β response in lung epithelial cells, which involves a type I IFN–positive feedback loop.

## Results

### RIG-I, TLR3 and NLRP3 contribute to the IL-1β response in primary lung epithelial cells infected by IAV

**Figure 1:**
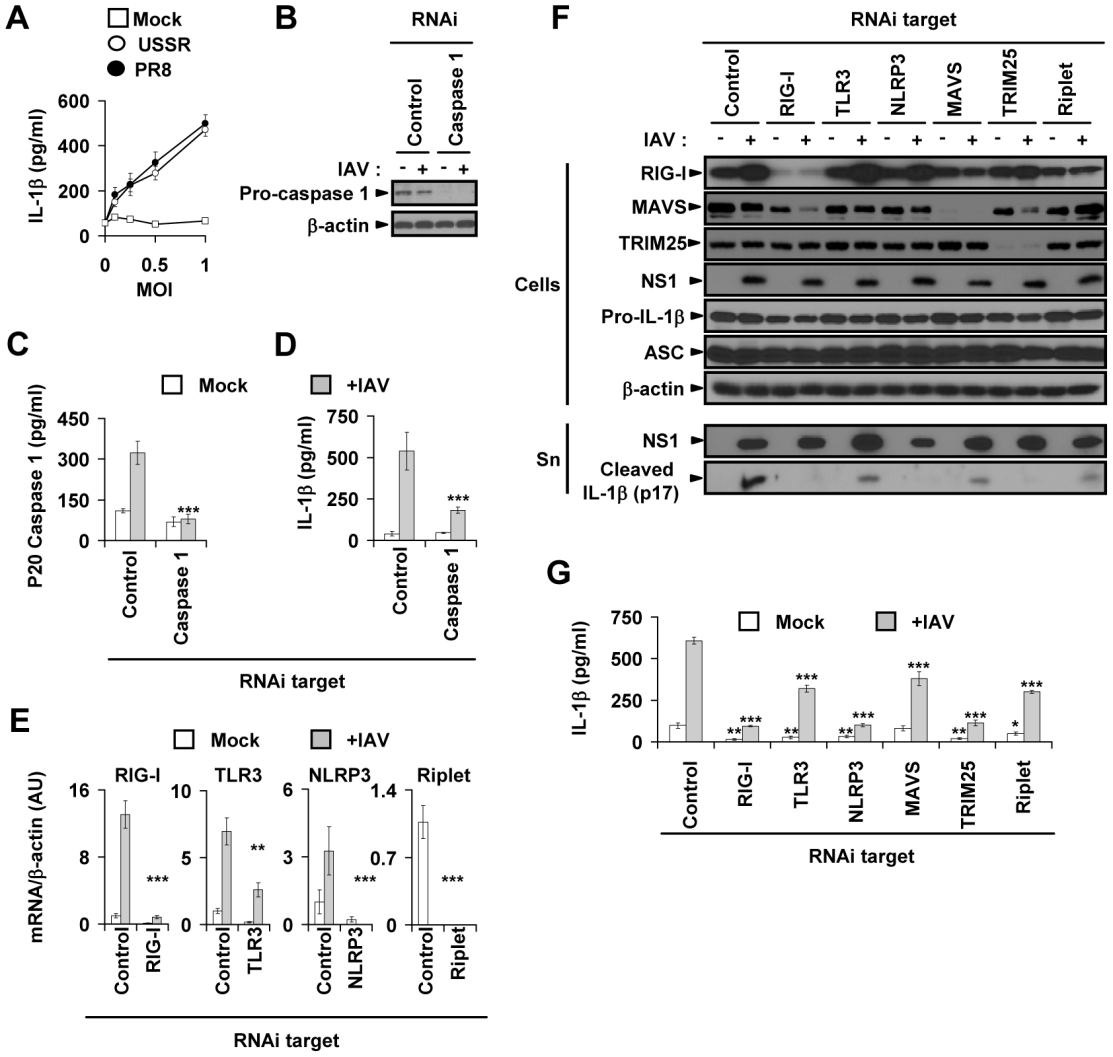
IAV infection triggers a RIG-I–, TLR3-, and NLRP3-dependent IL-1β response in primary lung epithelial cells. (A) Dose response of IL-1β production in mock-treated, as well as USSR-, and PR8-infected NHBE cells (*n* = 5 donors) at different MOIs for 18 h. From the supernatants of NHBE cells first either mock-treated (−) or infected with USSR (MOI 1, +IAV) and then transfected at 18 h post-infection with either control siRNA or siRNA targeting caspase I: (B) Pro-caspase I expression detected by immunoblot; ELISA quantification of (C) active caspase I p20 product, and (D) IL-1β. (E) RIG-I, TLR3, NLRP3, and Riplet mRNA expression in primary cells transfected with control or corresponding specific targeting siRNA before mock treatment or infection with USSR for 13 h. Data expressed as fold-increase relative to control siRNA and mock-treated cells (n = 4 donors). (F) NHBE cells were first transfected with either a control or an experimental siRNA, then either mock-treated (−) or infected with USSR (+). Samples were collected 18 h post-infection, on which an immunoblot analysis of intracellular RIG-I, MAVS, TRIM25, NS1, pro-IL-1β, ASC, and β-actin protein levels was performed; levels of cleaved IL-1β (p17) and secreted NS1 proteins were also assessed in the cell-free supernatants (Sn). The same membrane was probed with all specific antibodies. (G) ELISA quantification of IL-1β in the cell-free supernatants from the NHBE cells previously analyzed by immunoblot (Figure 1F). (F) and (G) respectively represent one out of two and one out of five experiments on different donors. *p<0.05, **p<0.01, and ***p<0.001, comparing mock-treated or IAV-infected cells transfected with specific siRNAs to their control siRNA-transfected cells counterparts.

**Figure 2:**
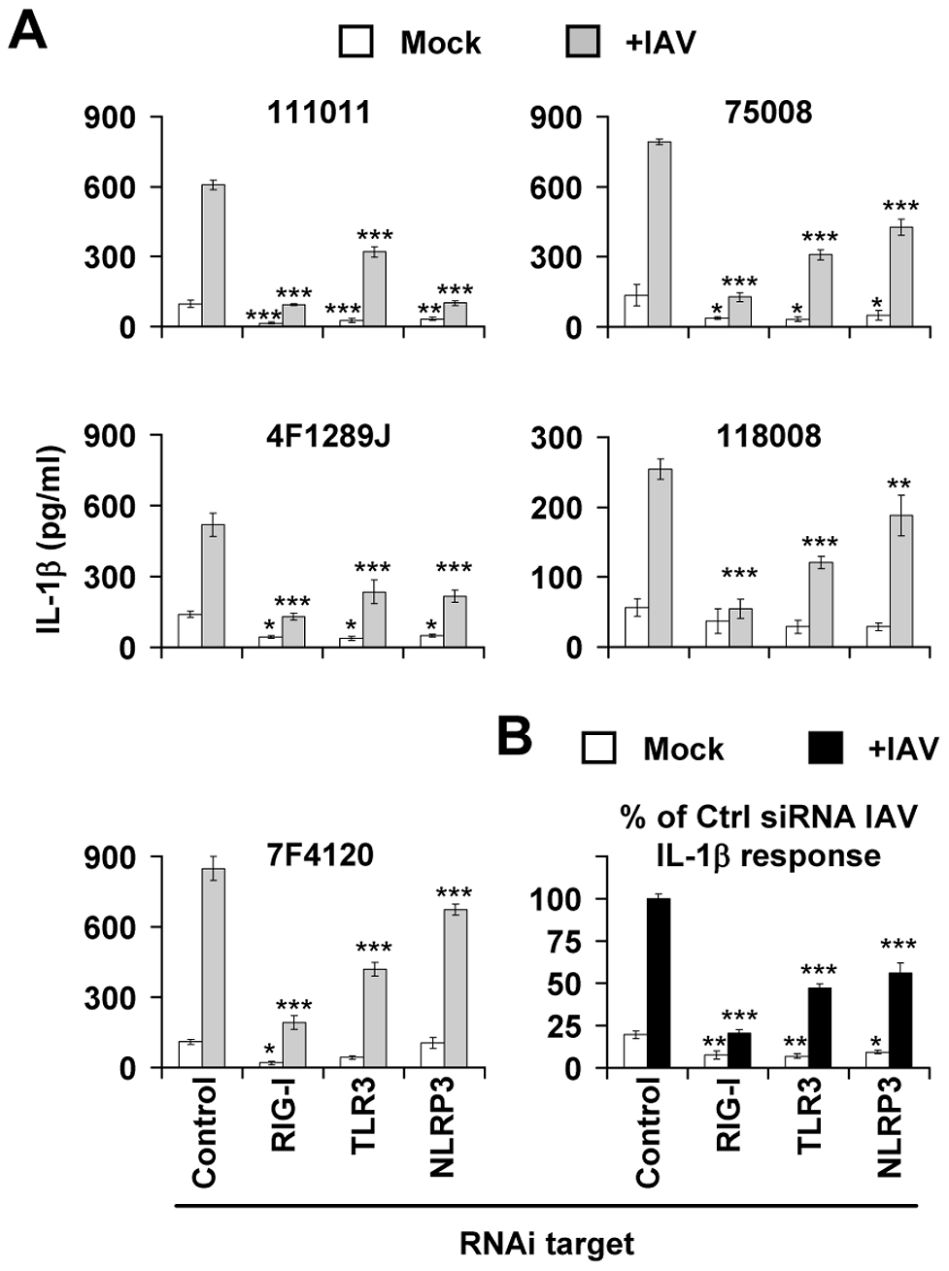
The IL-1β response depends on RIG-I, TLR3, and NLRP3 in primary NHBE cells from different donors. NHBE cells were isolated from five male donors (three of Caucasian origin [111011, 75008, 4F1289J] and two of African-American ancestry [118008, 7F4120]; see Table S1). These cells were then transfected with siRNA (either a control or one targeting either RIG-I, TLR3 or NLRP3) 18 h following either mock treatment or infection with USSR (+IAV). (A) IL-1β production in the cell-free supernatant of these cells. (B) Percentage of IL-1β production with respect to that of IAV-infected cells transfected with control siRNA. Data are presented as the mean ± SEM of the five experiments presented in (A), normalized with respect to control siRNA transfected cells infected with IAV (taken as 100%). *p<0.05, **p<0.01, and ***p<0.001, comparing mock-treated or IAV-infected cells transfected with specific siRNAs to their control siRNA-transfected cells counterparts.

To examine inflammasome activation in response to IAV infection in lung epithelial cells, we measured IL-1β production in normal, human primary bronchial epithelial cells (NHBE) derived from five donors (Table S1) following infection with the H1N1 viruses A/Puerto Rico/8/34 (PR8) and seasonal A/USSR/90/77 (USSR). Dose-response studies indicated that both virus strains induced IL-1β production in NHBE cells to similar levels (Figure 1A). Next, to study caspase 1 function in the IL-1β response to IAV in these primary cells, we knocked-down caspase 1 expression with specific siRNAs. As shown by western-blot for pro-caspase 1 (Figure 1B) and by ELISA quantification of p20 caspase 1 (Figure 1C), siRNA against caspase 1 almost abrogated pro-caspase 1 expression in IAV-infected and uninfected cells (Figure 1B), as well as p20 caspase 1 secretion in the cell supernatant of IAV-infected cells (Figure 1C). Consequently, we observed that the IL-1β response to IAV infection was dramatically decreased in cells transfected with siRNA against caspase 1 in comparison to cells transfected with control siRNA (Figure 1D). However, despite an efficient caspase 1 knockdown (Figures 1B and 1C), the remaining IL-1β secretion observed in response to IAV could suggest caspase 1-independent IL-1β production. These results demonstrate that caspase 1 has a critical role in the IL-1β response to IAV infection in NHBE primary cells. RIG-I, TLR3 and NLRP3 are expressed in lung epithelial cells [3], [4]. To characterize the mechanism underlying the IL-1β response in NHBE cells, we measured receptor transcript levels; we also assessed the efficiency of specific knockdowns of TLR3, RIG-I, and NLRP3 in primary NHBE cells at 13 h post-infection (time of peak transcriptional response to IAV). As expected, primary NHBE cells expressed the three PRRs, of which RIG-I showed the highest up-regulation in response to IAV infection (Figure 1E). Moreover, siRNA inhibition specifically and significantly reduced receptor transcript expression (Figure 1E) and RIG-I protein levels (Figure 1F), as well as the secretion of the active form of IL-1β (p17) into the supernatant (Figures 1F and G). When we measured NS1 protein levels on the same immunoblots, we did not detect a decrease in intracellular or supernatant NS1 levels of cells treated with specific siRNAs compared to those treated with control siRNA (Figure 1F). This demonstrates that siRNA treatment did not prevent or attenuate infection. The results were confirmed in USSR- and PR8-infected cells from 3–5 donors (Figures 2A and B and S1), further indicating that the observed changes in IL-1β secretion were PRR-dependent. We then examined in more detail the function of individual PRRs in NHBE cells. Among the PRRs tested, RIG-I downregulation had the strongest effect, resulting in a 3.1-fold and a 3.7-fold greater inhibition of IL-1β secretion than TLR3 and NLRP3 siRNA treatments, respectively, in both USSR- and PR8-infected primary NHBE cells (Figures 1F and G; 2B; and S1A, S1C and S1D; *p*<0.0001). The role of RIG-I was further studied using siRNAs to knock down the expression of its signaling partners: MAVS, TRIM25 [18] and Riplet [19]. Downregulation of each of these transcripts resulted in a significant inhibition of the secretion of cleaved IL-1β upon IAV infection (Figures 1F and G). Altogether, these data indicate that IAV infection triggers a potent IL-1β response in human primary lung epithelial cells. This response involves RIG-I, TLR3, and NLRP3; RIG-I appeared to have the strongest effect, through a MAVS/TRIM25/Riplet–dependent signaling pathway.

### RIG-I is a pivotal regulator of inflammasome activation in IAV-infected primary lung epithelial cells

**Figure 3:**
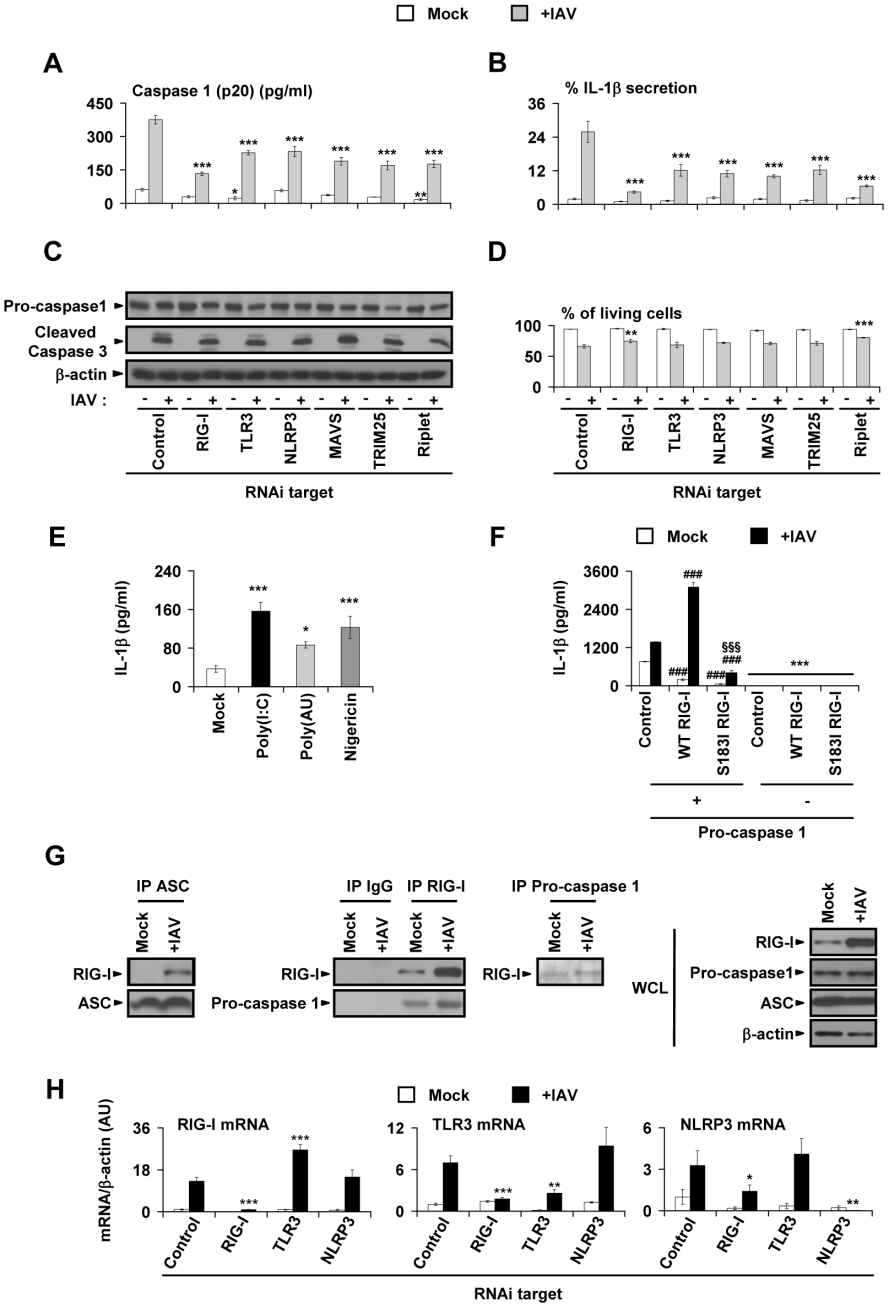
RIG-I-dependent caspase 1 activation is critically involved in IL-1β response to IAV. (A–D) NHBE cells were first transfected with either a control or one of the experimental siRNA, and then either mock-treated (−) or infected with USSR (+IAV). At 18 h post-infection or mock treatment, the following were measured in the cell-free supernatant: (A) Cleaved caspase 1 p20; (B) Ratio of secreted IL-1β normalized with respect to total IL-1β production (intracellular and secreted IL-1β), expressed as a percentage (concentration measurements are provided in supplementary Tables S2 and S3). (C) Immunoblot detection of pro-caspase 1, cleaved caspase-3, and β-actin expression. (D) Percentage of living NHBE cells determined by LDH assay (calculation is detailed in the supplemental experimental procedures file). (A–D) Experiments were performed on 3–5 NHBE donors, *p<0.05, **p<0.01, and ***p<0.001, comparing mock-treated or IAV-infected cells transfected with specific siRNAs to their control siRNA-transfected counterparts. (E) ELISA quantification of IL-1β in the cell-free supernatant derived from NHBE cells following 22 h of either mock-treatment or stimulation with either the TLR3 ligands Poly(I:C) (5 µg/ml) or Poly(AU) (100 µg/ml), or the NLRP3 ligand nigericin (10 µM). The data are representative of three independent experiments and presented as mean ± SEM of triplicates with cells from three donors (75008, 118008, and 4F1289J). *p<0.05, **p<0.01, and ***p<0.001, stimulated cells compared with mock-treated cells. (F) HEK 293T cells were first transfected with ASC and pro-IL-1β expression vectors, along with: 1) either an empty control vector or FLAG-WT RIG-I or FLAG-S183I RIG-I expression vectors; and 2) either an empty control vector (−) or a pro-caspase 1 (+) expression vector. The cells were then either mock-treated or infected with PR8 (+IAV). (F) shows IL-1β expression in these cells following mock treatment or infection. ***p<0.001, comparing (+) caspase 1 with (−) caspase 1; ###p<0.001, WT or S183I RIG-I compared with empty vector (control). §§§p<0.001, WT compared with S183I RIG-I. (G) Endogenous interactions of RIG-I with ASC and pro-caspase 1 were assessed in NHBE cells, 18 h after mock or USSR infection (+IAV, MOI 1). ASC, RIG-I, pro-caspase 1 were immunoprecipitated (IP ASC, IP RIG-I, IP Pro-caspase 1) and immunoblotted (ASC, RIG-I, Pro-caspase 1). As a negative control of immunoprecipitation anti-FLAG antibody was used (IP IgG). WCL: immunoblots on whole cell lysates. (H) NHBE cells were first transfected with a control or experimental siRNA, and then either mock-treated or infected with USSR (+IAV). (H) shows RIG-I, TLR3, and NLRP3 mRNA expression at 13 h post-infection or mock treatment. Data are presented as a mean fold increase ± SEM of mRNA expression relative to control siRNA and mock-treated cells, derived from eight qRT-PCRs, spread over four independent experiments and using cells from four donors, normalized to β-actin levels.*p<0.05, **p<0.01, and ***p<0.001, compared mock-treated or IAV-infected cells transfected with specific siRNAs to their control siRNA-transfected cells counterparts.

To further investigate the mechanisms underlying the IL-1β response to IAV infection in primary lung epithelial cells, we studied the contribution of each PRR to the levels of intracellular or activated forms of IL-1β, ASC, and caspase 1. During IAV infection, the siRNA transfection procedure does not affect the pro-IL-1β protein level: untransfected cells and those transfected with control siRNA display similar levels of the protein (Figure S2A). Furthermore, intracellular pro-IL-1β protein levels in epithelial cell samples treated with either experimental or control siRNAs were not notably different between mock-treated and IAV-infected cells (Figure 1F). However, the level of activated caspase 1 (p20) in the supernatant (Figure 3A) and the percentage of IL-1β secretion, which corresponds to secreted IL-1β normalized with respect to both intracellular and secreted IL-1β (Figure 3B, see Tables S2 and S3 for measured concentrations), were significantly reduced by downregulation of RIG-I; TLR3; NLRP3; or the RIG-I-signaling partners MAVS, TRIM25, and Riplet. The stable pro-caspase 1 observed in all samples (Figures 3C and S2B) illustrates that these siRNAs specifically inhibited caspase 1 activation. ASC expression was similar in all experimental conditions (Figure 1F), which indicates that the effect of siRNA on caspase 1 activation is ASC-independent. To rule out cell death as the cause for the observed differences in IL-1β secretion, we determined the levels of caspase 3 cleavage, a common marker of apoptosis, by immunoblot and quantified the proportion of living cells by LDH assay, a measurement of cell membrane integrity. We did not observe any differences in cleaved caspase 3 levels (Figure 3C), LDH activity (Figure 3D), or cell count (Figure S2C) between IAV-infected samples treated with either control or experimental siRNAs. Taken together, these results indicate that TLR3 and NLRP3, as well as RIG-I and its downstream signaling partners MAVS, Riplet, and TRIM25, elicit inflammasome activation in response to IAV infection by regulating caspase 1 activation and, consequently, IL-1β secretion in a manner independent of cell death. In support of our findings that TLR3 and NLRP3 play a role in inflammasome activation, we found that stimulation of NHBE cells with specific agonists – the synthetic dsRNAs poly(I:C) and poly(A:U) for TLR3, and the pore-forming microbial toxin nigericin for NLRP3 – resulted in significant IL-1β secretion (Figure 3E). To better characterize the role of RIG-I in the caspase 1–dependent IL-1β response to IAV infection, we developed a HEK 293T reporter cell assay in which the different components required for IL-1β activation (ASC, pro-caspase 1, and pro-IL-1β) are produced from expression vectors in the presence or absence (empty vector–transfected cells) of wild-type (WT) RIG-I protein. As a negative control, we used S183I, a loss-of-function variant of RIG-I that is unable to trigger antiviral and pro-inflammatory responses to IAV [25]. S183I affects the second CARD domain of the RIG-I protein. We previously used a combination of biochemical, functional and structural modeling analyses to establish that the single nucleotide polymorphism (SNP) S183I inhibits RIG-I-dependent signaling by stabilizing hydrophobic-interaction between CARD domains [25]. Here we observed that, in the presence of WT RIG-I protein, there was a significant upregulation of the IL-1β response to IAV PR8 (Figure 3F; similar results were obtained upon USSR infection, data not shown) compared to cells transfected with the empty vector. This IL-1β response was significantly inhibited in the absence of pro-caspase 1 (Figure 3F) or in the presence of S183I (Figure 3F) relative to control cells and WT RIG-I transfected cells. To confirm that RIG-I can directly activate the inflammasome through its binding to caspase 1 and ASC, we performed coimmunoprecipitation assays of either caspase 1 or ASC with RIG-I in primary lung epithelial cells. Notably, while the levels of expressed and immunoprecipitated ASC protein were similar in mock-treated and IAV-infected NHBE cells (Figure 3G, WCL and IP ASC), RIG-I/ASC complexes were detectable only in IAV-infected cells (Figure 3G, IP ASC). Endogenous RIG-I also interacts with caspase 1. Indeed, caspase 1 coimmunoprecipitated with RIG-I, when either RIG-I (IP RIG-I) or caspase 1 (IP caspase 1) were immunoprecipitated, but not when we used a control antibody (IP IgG) (Figure 3G). By contrast with ASC, caspase 1 interacted with RIG-I in both uninfected and IAV-infected cells; the number of complexes was correlated with RIG-I expression, with the highest amount of complexes in IAV-infected cells. These results demonstrate that a functional RIG-I receptor is required for caspase 1–dependent inflammasome activation in response to IAV infection of lung epithelial cells. In response to IAV infection, RIG-I interacts with ASC, and the number of RIG-I/caspase 1 complexes is increased. Thus RIG-I could directly activate the ASC/caspase 1 inflammasome in primary lung epithelial cells. Finally, to investigate possible cross-regulation between PRRs during IAV infection, we evaluated RIG-I, TLR3, and NLRP3 transcript expression in IAV-infected primary human lung epithelial cells treated with siRNAs against each PRR (Figure 3H). In the context of IAV infection, RIG-I expression was not affected by TLR3 or NLRP3 downregulation, while TLR3 and NLRP3 expression were significantly inhibited in RIG-I knockdown cells (Figure 3H). In contrast, overexpression of RIG-I significantly increased NLRP3 and TLR3 expression in both NHBE primary cells and HEK 293T cells (Figure S3). RIG-I overexpression also potentiated TLR3 up-regulation in response to IAV infection (Figure S3). These results indicate that TLR3 and NLRP3 transcript expression is RIG-I–dependent in lung epithelial cells. Furthermore, optimal IL-1β production in primary lung epithelial cells requires RIG-I–mediated TLR3 and NLRP3 upregulation.

### Type I IFNs are positive regulators of the RIG-I–dependent IL-1β response to IAV

**Figure 4:**
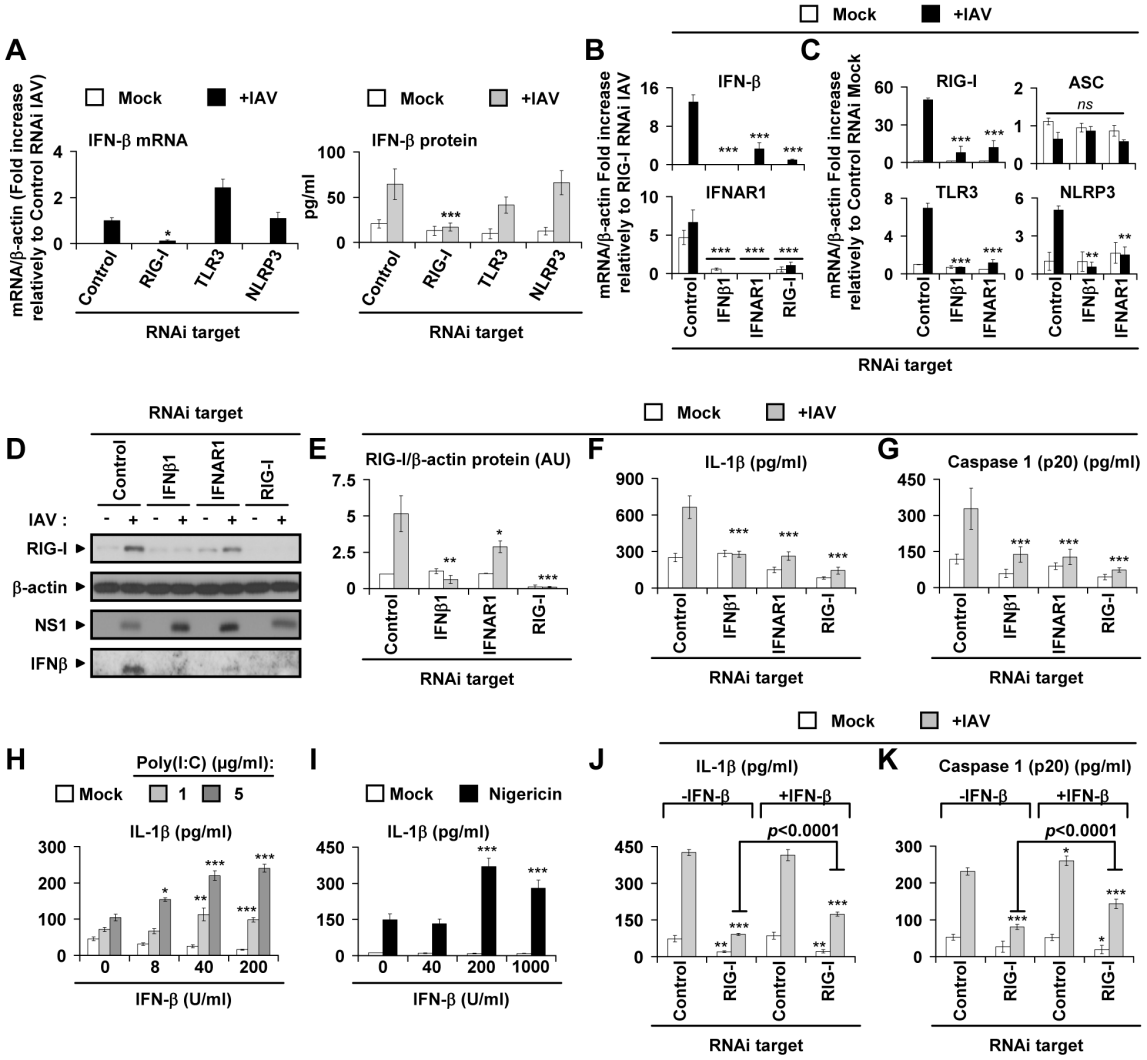
IL-1β response to IAV depends on type I IFN–mediated RIG-I signaling. (A) NHBE cells were first transfected with either control or RIG-I-, TLR3-, or NLRP3–targeting siRNAs, before mock treatment or infection with USSR (+IAV) for 13 h. *First panel*. IFN-β mRNA expression. Data are presented as mean ± SEM mRNA expression, derived from eight qRT-PCRs, spread over four independent experiments and using cells from four donors, normalized with respect to β-actin level. Results are expressed as fold-increase relative to control siRNA transfected cells infected with IAV. *Second panel*. IFN-β protein production in the cell-free supernatant of these cells. (B) NHBE cells were transfected with control or IFN-β-, IFNAR1-, or RIG-I–targeting siRNAs, followed by mock treatment or USSR (+IAV) infection for 18 h. IFN-β and IFNAR1 expression was quantified by qRT-PCR. (C) RIG-I, TLR3, ASC and NLRP3 expression in NHBE cells transfected with control siRNA or siRNA targeting IFN-β or IFNAR1. Data are presented as a fold-increase relative to RIG-I siRNA-transfected and IAV-infected cells (B) or control siRNA-transfected and mock-treated cells (C). (D) Immunoblot of RIG-I, β-actin, NS1 and IFN-β in NHBE cells stimulated as in (B). (E) Ratio of RIG-I protein signal on immunoblots normalized to the β-actin signal. Results are presented as a mean fold increase ± SEM relative to mock-treated cells transfected with control siRNA. (F) IL-1β and (G) cleaved caspase 1 p20 production in the cell-free supernatant of NHBE cells stimulated as in (B). (A–G) *p<0.05, **p<0.01, and ***p<0.001, comparing cells transfected with a specific siRNA to their control siRNA transfected counterparts. (H and I) NHBE cells were pretreated with various amounts of recombinant human IFN-β for 24 h (H) or 6 h (I) before a 22 h challenge with poly(I:C) (H) or nigericin (I). IL-1β secretion was quantified by ELISA in the cell-free supernatants. *p<0.05, **p<0.01, and ***p<0.001, comparing IFN-β pretreated with untreated cells for a given stimulation condition. (J and K) NHBE cells were transfected with either control or RIG-I-targeting siRNA and then infected with either mock or USSR (+IAV MOI 1) for 8 h before treatment with either IFN-β (+IFN-β) or mock (−IFN-β). (J) IL-1β and (K) cleaved caspase 1 p20 production in the cell-free supernatant. (J and K) *p<0.05, **p<0.01, and ***p<0.001 relative to control siRNA-transfected cells without IFN-β treatment in the same experimental condition (mock or +IAV respectively). *p*<0.0001 between IFN-β-treated (+IFN-β) and untreated (−IFN-β) cells transfected with RIG-I-targeting siRNA and infected with IAV.

During IAV infection, RIG-I is a critical regulator of type I IFNs in lung epithelial cells [4], [5]; its expression is amplified by a positive feedback loop via type I IFNs [4]. In primary NHBE cells, knockdown of RIG-I, but not of TLR3 or NLRP3, almost completely abrogated the IFN-β response at both the mRNA and the protein level (more than 75% of inhibition, Figure 4A); this confirms the pivotal role of RIG-I in the type I IFN response to IAV in these cells. The NLRP3 promoter sequence contains predicted binding sites for IFN regulatory elements (data not shown), suggesting that type I IFNs regulate the expression of this receptor, as reported for RIG-I and TLR3 in lung epithelial cells [4], [5], [26]. To address this possibility, we used specific siRNAs to knock down the expression of RIG-I, IFN-β, and the type I IFN receptor 1 alpha chain (IFNAR1) in NHBE cells. These siRNA treatments successfully abrogated IFN-β and IFNAR1 transcript expression (Figure 4B). They also severely reduced transcript levels of RIG-I, TLR3, and NLRP3 (Figure 4C) and significantly decreased RIG-I and IFN-β protein levels (Figures 4D–E and S4A), as compared to cells transfected with control siRNA. By contrast, ASC and caspase 1 expression were not affected in the same samples (Figures 4C and S4B in Text S1). Concomitant with these changes, we observed increased NS1 protein (Figures 4D and S4C) levels when IFN-β and IFNAR1 expression were completely inhibited, indicating uncontrolled IAV replication, as expected in the absence of type I IFN responses (Figure 4B). In contrast, the NS1 level was not significantly different between RIG-I and control knockdown cells which could be due to the remaining IFN-β signaling in these cells (Figure 4B). Importantly, inhibition of the type I IFN axis also resulted in a >95% reduction of IL-1β and cleaved caspase 1 secretion in response to IAV infection (Figures 4F and G). To confirm the role of type I IFN in inflammasome activation, NHBE cells were pretreated with recombinant IFN-β before being challenged with the ligands of either TLR3 (poly(I:C), Figure 4H) or NLRP3 (nigericin, Figure 4I). We observed that IFN-β pretreatment enhanced the IL-1β response to poly(I:C) and nigericin challenge (Figures 4H and I) in a dose-dependent manner, indicating that RIG-I–dependent type I IFN induces inflammasome activation by increasing RIG-I, TLR3, and NLRP3 expression in IAV-infected primary lung epithelial cells. Since RIG-I plays a pivotal role in IFN-β expression, it thus regulates TLR3 and NLRP3 expression. This positive feedback loop could play a critical role in RIG-I-dependent inflammasome activation. As described above, RIG-I directly interacts with ASC and caspase 1. To functionally address RIG-I-dependent, but type I IFN-independent, inflammasome activation, RIG-I expression was either inhibited or not (control) by siRNA transfection in primary NHBE cells. Cells were mock-treated or infected with IAV 8 h before treatment with exogenous IFN-β. Notably, when RIG-I was knocked-down, IFN-β treatment significantly increased IL-1β and p20 caspase 1 secretion in the context of IAV infection (p<0.0001, Figures 4J and 4K). However, as expected if RIG-I has a direct effect on inflammasome activation, IFN-β treatment was not able to restore complete IL-1β secretion and caspase 1 activation in RIG-I knockdown cells (p<0.0001, Figures 4J and 4K). Hence, these data demonstrate the critical role of RIG-I in a type I IFN positive feedback loop that regulates RIG-I, TLR3, and NLRP3 up-regulation. Moreover these results support the hypothesis of a functional role for RIG-I in direct inflammasome activation in IAV infected cells, as suggested by ASC and caspase 1 interaction with RIG-I shown in Figure 3G.

### NS1-mediated inhibition of the IL-1β response correlates with higher virulence in ferrets

**Figure 5:**
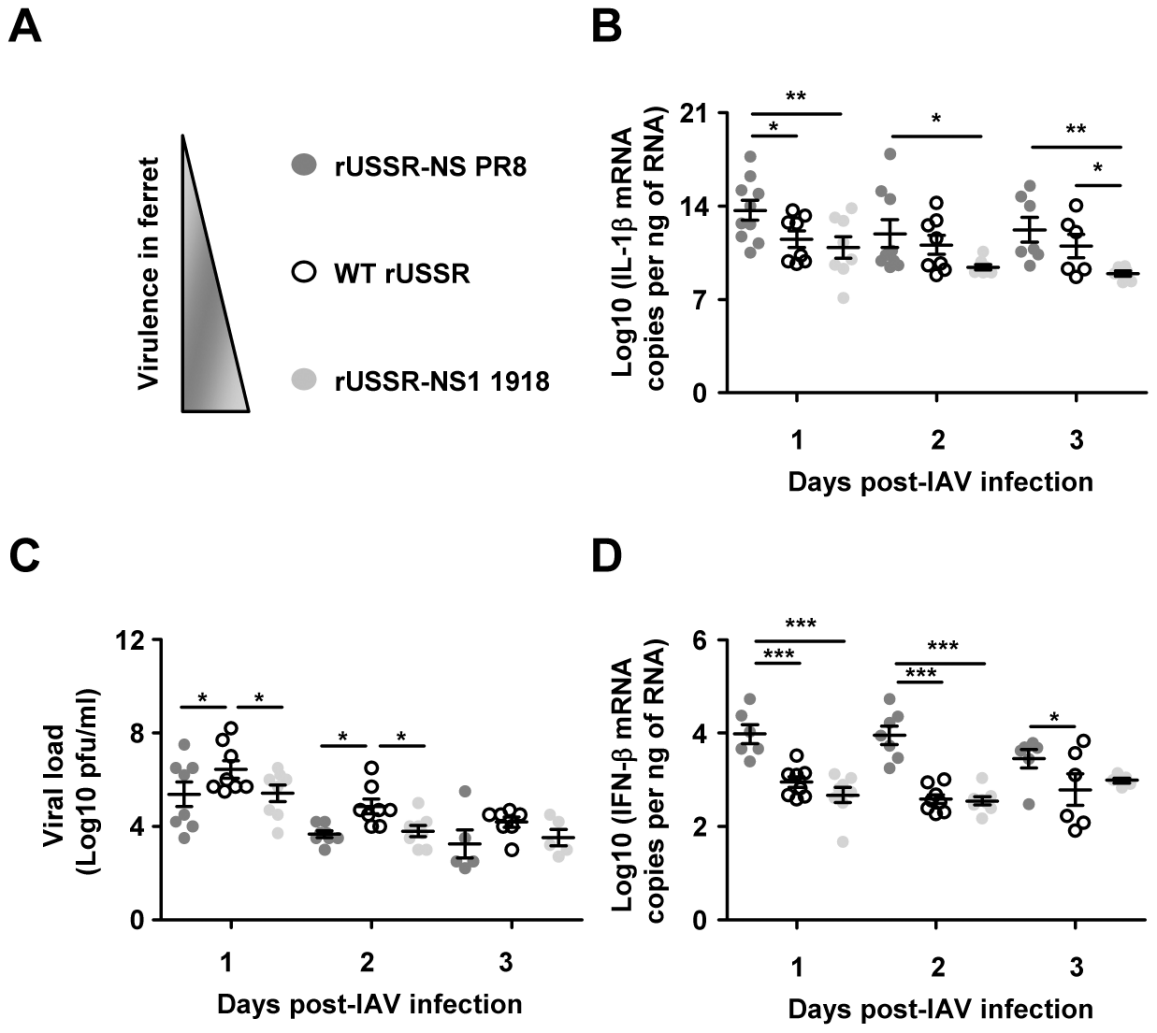
1918 NS1 inhibits IL-1β response and type I IFN response, and results in higher virulence *in vivo* in ferrets. (A) Schematic representation of pathogenesis upon infection with rUSSR-NS1 1918, rUSSR-NS PR8, or WT rUSSR, as previously described [20]. (B) Kinetics of IL-1β mRNA response to rUSSR-NS PR8 (*n* = 10), WT rUSSR (*n* = 8), and rUSSR-NS1 1918 (*n* = 8) in cells from nasal wash of infected ferrets at days 1, 2, and 3 post-infection. (C) Viral load in nasal wash from the same ferrets as in (B) presented as a log10 of pfu/ml. (D) Kinetics of IFN-β mRNA response to rUSSR-NS PR8, WT rUSSR, and rUSSR-NS1 1918 under the same experimental conditions as (B). (B and D) Data presented as a log10 of IL-1β or IFN-β mRNA copy number per nanogram of RNA with mean ± SEM: calculated from a standard curve generated from *in vitro*–transcribed mRNA of known concentration. *p<0.05, **p<0.01, and ***p<0.001.

NS1 is the main IAV IFN antagonist [2], [18], [20]–[23]. Our finding that type I IFN is a positive regulator of IL-1β in lung epithelial cells led us to investigate the impact of NS1 on the IL-1β host response. In a previous study using reassortant USSR viruses bearing the NS1 protein of either moderately virulent USSR (WT rUSSR), attenuated PR8 (rUSSR-NS PR8), or highly virulent 1918 (rUSSR-NS1 1918) H1N1 strains, we observed that NS1-mediated inhibition of IFN induction correlates with the virulence of the respective virus in ferrets [20]. rUSSR-NS PR8 was used instead of rUSSR-NS1 PR8, since replication of the latter virus was impaired *in vitro* in an earlier study [20]. To examine the relationship between NS1-mediated inhibition of type I IFN and IL-1β responses *in vivo*, we quantified IL-1β and IFN-β transcript levels in nasal wash cells from ferrets infected with the different viruses in the context of an earlier study [20]. The most severe clinical signs - which are represented by the sum of scores attributed to upper and lower respiratory signs, breathing rate, activity and endurance, as well as lung pathology - were observed for rUSSR-NS1 1918, followed by WT rUSSR, while rUSSR-NS PR8 caused the mildest disease ([20] and Figure 5A). Here, we observed an increase in IL-1β expression, although with significant inter-virus differences, in which rUSSR-NS1 1918 induced the lowest IL-1β response (Figure 5B). Notably, at days 1 to 3, rUSSR-NS1 1918 infection resulted in a significantly lower IL-1β response than rUSSR-NS PR8 infection (Figure 5C), even though viral load was similar between the two groups of infected ferrets (Figure 5C). At day 3, viral load was also similar in WT rUSSR- and rUSSR-NS1 1918-infected ferrets (Figure 5C); yet rUSSR-NS1 1918 infection resulted in a significantly lower IL-1β response than WT rUSSR infection (Figure 5B). These data suggest that the 1918 NS1 protein results in the most potent inhibition of the ferret IL-1β response *in vivo*. Furthermore, consistent with the association of type I IFN and IL-1β responses described above, in these experiments, reduced IL-1β expression levels were positively correlated with reduced IFN-β expression levels at days 1 and 2 post-infection (Figures 5B and 5D, day 1: r = 0.56, p = 0.0063; day 2: r = 0.47 and p = 0.0235). By contrast to rUSSR-NS PR8, which was not detectable at day 2 and 4 post-infection in the lungs, USSR and 1918 NS1 protein inhibition of type I IFN and IL-1β responses were associated with sustained replication and spread to the lungs ([20] and Figures 5B and 5D). In mice, increased IL-1β production in response to IAV infection was associated with reduced histopathology [27]. Here we noted that rUSSR-NS PR8 infection, which resulted in the highest IL-1β expression (Figure 5B), produced the least tissue damage [20]. This suggests that IL-1β protein expression is proportional to its mRNA expression pattern, at least for rUSSR-NS PR8-infected ferrets as compared to WT rUSSR and rUSSR-NS1 1918 infected ferrets. Due to the lack of IL-1β assay in ferrets we were unable to further investigate NS1 impact on IL-1β response *in vivo* or *ex vivo* in ferrets. However, the well described inhibitory effect of NS1 on the type I IFN response [2], [18], [20]–[23], together with our *ex vivo* results with primary NHBE cells showing that IFN-β is critical to IL-1β response, led us to study NS1 impact on RIG-I/type I IFN/IL-1β signaling in correlation with the known virulence *in vivo* in ferret.

### 1918 NS1 inhibits type I IFN–dependent RIG-I upregulation and subsequently IL-1β secretion

**Figure 6:**
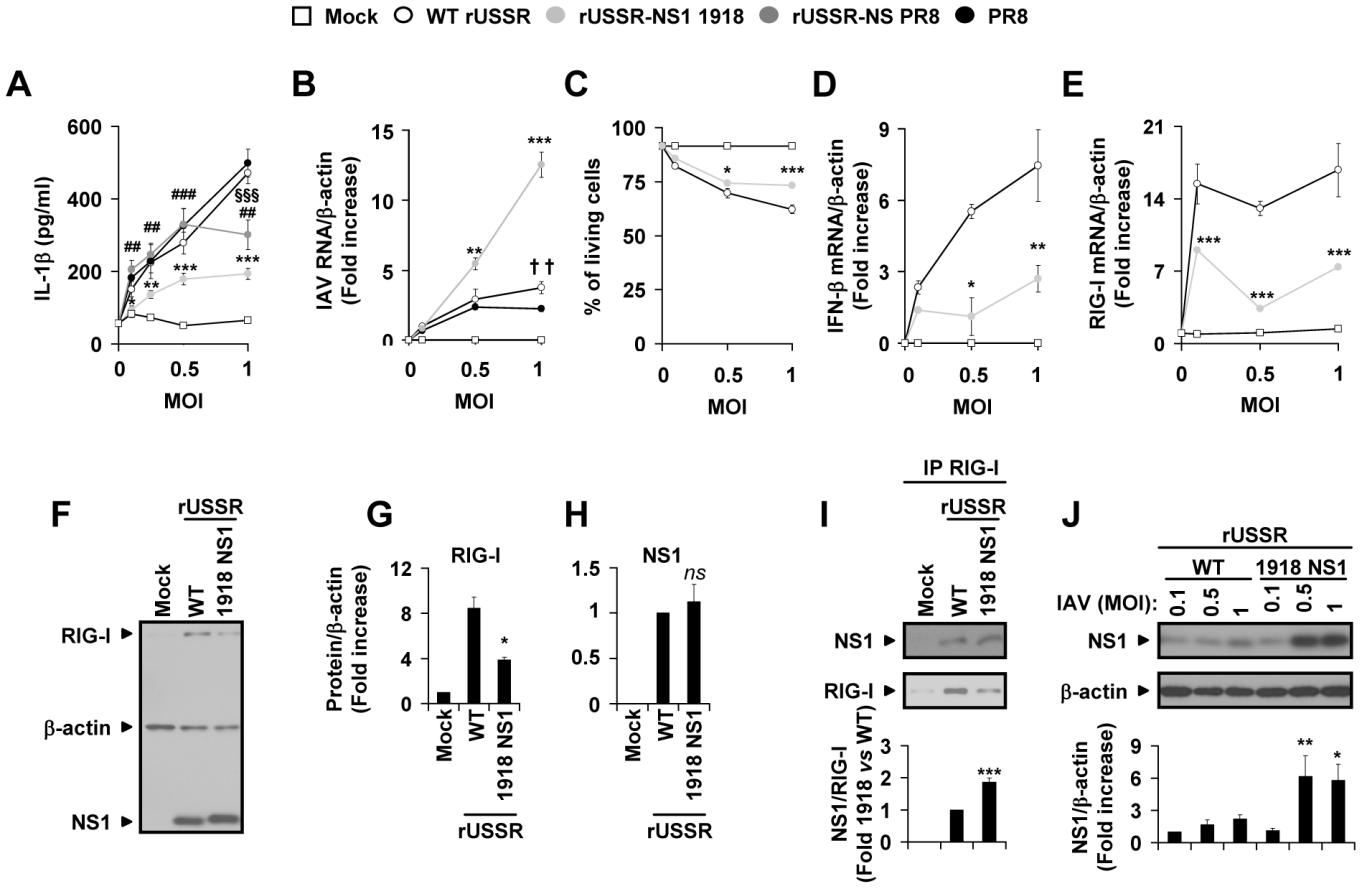
The pandemic virus 1918 NS1 inhibits IL-1β response and downregulates type I IFN–mediated RIG-I upregulation. (A) NHBE cells from five donors were either mock-treated or infected for 18 h with WT PR8, WT rUSSR, rUSSR-NS1 1918, or rUSSR-NS PR8 at various MOIs. IL-1β production in cell-free supernatants was quantified by ELISA. (B) Virus M2 RNA levels normalized with respect to β-actin levels. (C) Percentage of living cells determined by LDH assay. (D) IFN-β and (E) RIG-I mRNA levels normalized with respect to β-actin levels. (B, D and E) Data are presented as a mean ± SEM of quadruplicates of qRT-PCR of two experiments on NHBE cells from donor 111011. Results are expressed as fold-increase relative to WT rUSSR-infected cells at an MOI of 0.1 (B and D) and relative to unstimulated cells (E). (F–H) NHBE cells were either mock-treated or infected with either rUSSR WT or NS1 1918 for 18 h. (F) Immunoblot of RIG-I, NS1, and β-actin. Quantification of (H) the RIG-I/β-actin and (G) the NS1/β-actin protein signal ratios. (I) Endogenous interactions between RIG-I and either USSR or 1918 NS1 proteins were assessed in NHBE cells stimulated as in (F). RIG-I was immunoprecipitated with anti-RIG-I antibody (IP RIG-I) and immunoblotted (RIG-I). NS1 interaction with RIG-I was analysed by immunoblotting NS1 (NS1) after RIG-I immunoprecipitation and The NS1/RIG-I protein signal ratio in immunoprecipitated samples was quantified. (J) Immunoblot of β-actin, USSR and 1918 NS1 proteins in NHBE cells infected with multiple MOI of recombinant viruses for 18 h. The NS1/β-actin protein signal ratios in these NHBE cells are presented on the bottom panel. (F to J) One representative immunoblot out of three is presented. Results are presented as a mean ± SEM of immunoblots from three independent experiments (G, I and J) and from six independent experiments (H). Data are expressed as a fold increase relative to mock-treated cells (G), WT rUSSR-infected cells (H and I) or WT rUSSR infected-cells at an MOI of 0.1 (J). (A–I) *p<0.05, **p<0.01, and ***p<0.001 NS1 1918 compared with WT rUSSR infected cells. #*p*<0.05, ##*p*<0.01, and ###*p*<0.001 NS PR8 compared with NS1 1918 rUSSR. §*p*<0.05, §§*p*<0.01, and §§§*p*<0.001 NS PR8 compared with WT rUSSR. On panels A and B, *p<0.05, **p<0.01, and ***p<0.001 also comparing NS1 1918 with PR8. †† *p*<0.01 comparing WT rUSSR with PR8.

To investigate if NS1 proteins from different strains vary in their interference with the IL-1β response observed in lung epithelial cells, we quantified IL-1β secretion in NHBE cells (from five different donors) after infection with different multiplicities of infection (MOI) of the NS1 recombinant and parental viruses. Infection with recombinant rUSSR-NS1 1918 resulted in the lowest IL-1β levels in NHBE cells compared to infection with parental WT rUSSR, PR8, and rUSSR-NS PR8 (Figure 6A). The extent of IL-1β secretion did not correlate with viral growth, which was not different at a low MOI (MOI 0.1) and significantly higher for rUSSR-NS1 1918 than for parental WT rUSSR and PR8 in NHBE cells infected at an MOI of 0.5 and 1 (Figure 6B). Kinetics analyses of infection with rUSSR-NS1 1918 and WT rUSSR M2 RNA in a human lung epithelial cell line (NCI-H292) indicated that the differences in viral growth observed at a high MOI do not seem to be due to differences in virus infection level. Indeed, M2 RNA derived from both viruses are similarly expressed at early time-points post-infection (3 h, 6 h, and 9 h, Figure S5). By contrast, rUSSR-NS1 1918 infection resulted in significantly higher levels of virus M2 RNA at later time-points (13 h and 18 h, Figure S5). Because 1918 NS1 inhibited IL-1β secretion, we focused on rUSSR-NS1 1918 and WT rUSSR strains to specifically study the impact of NS1 on the IL-1β response in lung epithelial cells. The differential IL-1β response was correlated with cell viability (r = −0.903, p<0.0001) but seems only slightly dependent on cell death, since a less than 11% difference in live cells was observed in NHBE cells infected with WT rUSSR or rUSSR-NS1 1918 (Figure 6C). More importantly, as observed earlier, the amount of secreted IL-1β correlated with the significantly lower levels of type I IFN (Figure 6D, r = 0.885, p<0.0001) and RIG-I (transcript r = 0.593, p = 0.0327, Figure 6E; protein, Figure 6F and G) expressed during infection with rUSSR-NS1 1918 compared to rUSSR. Moreover, this correlation was also independent of viral replication and thus of NS1 protein expression at a low MOI (MOI 0.1, Figure 6B, F and H). Finally, coimmunoprecipitation experiments, performed at a low MOI, when NS1 protein levels are similar, suggested that 1918 NS1 formed more prominent complexes with RIG-I than USSR NS1 (Figure 6I). As NS1 binding to RIG-I has been shown to inhibit RIG-I-dependent signaling [21], increased binding of 1918 NS1 may contribute to its potent inhibition of type I IFN signaling and RIG-I expression. However, NS1 is a multi-functional protein that interacts with multiple cellular factors to inhibit the host type I IFN response [2], [18], [20]–[23]. Thus the inhibitory impact of 1918 NS1 could be due to its impact on RIG-I activity, through direct protein-protein interactions or indirectly by regulating the type I IFN signaling downstream of RIG-I thereby interfering with the type I IFN-dependent RIG-I upregulation. Furthermore the higher replication of 1918 NS1 at a MOI more than 0.1 (Figure 6B) is associated with higher expression of 1918 NS1 protein (Figure 6J), which may amplify the inhibitory activity of 1918 NS1. In support to this hypothesis, the type I IFN response, which is RIG-I-dependent, was significantly lower at MOIs of 0.5 and 1 (Figure 6D). The lower type I IFN response could be due to the increased inhibitory effect of NS1 on RIG-I or other type I IFN signaling components downstream of RIG-I and may explain the higher viral replication observed in these conditions (Figure 6B). These results indicate that the IL-1β response to IAV infection in lung epithelial cells is regulated by NS1, probably through its inhibitory action on the type I IFN positive feedback loop required for RIG-I upregulation; they also confirm the potent inhibitory activity of 1918 NS1. Thus, NS1 from the highly pathogenic human pandemic virus 1918, which most strongly binds to RIG-I and thus results in the strongest inhibition of type I IFN signaling in NHBE cells, also induces the strongest inhibition of IL-1β in NHBE cells, and of the IL-1β and type I IFN responses in ferrets. As IAV-triggered immunopathogenesis in ferrets is very closed to that in humans, one could speculate that the high virulence of rUSSR-NS1 1918, associated with the lowest type I IFN expression in ferrets, could be due to inhibitory impact of NS1 on RIG-I/type I IFN/IL-1β signaling as we demonstrated in human primary lung epithelial cells.

## Discussion

IL-1β plays a beneficial role in the *in vivo* innate immune response to IAV infection [3], [27], [28]. Our findings reveal a novel regulation of the inflammasome in lung epithelial cells, which are both the primary target of IAV infection *in vivo* and major players in the outcome of infection [4]–[9]. In IAV-infected macrophages, IL-1β secretion is NLRP3-, ASC-, and caspase 1-dependent; it has also been suggested to be RIG-I-independent [3], [11]. Conversely, in primary human lung epithelial cells, RIG-I, TLR3, and NLRP3 are partially redundant for IL-1β activation through a caspase 1–dependent mechanism.

**Figure 7:**
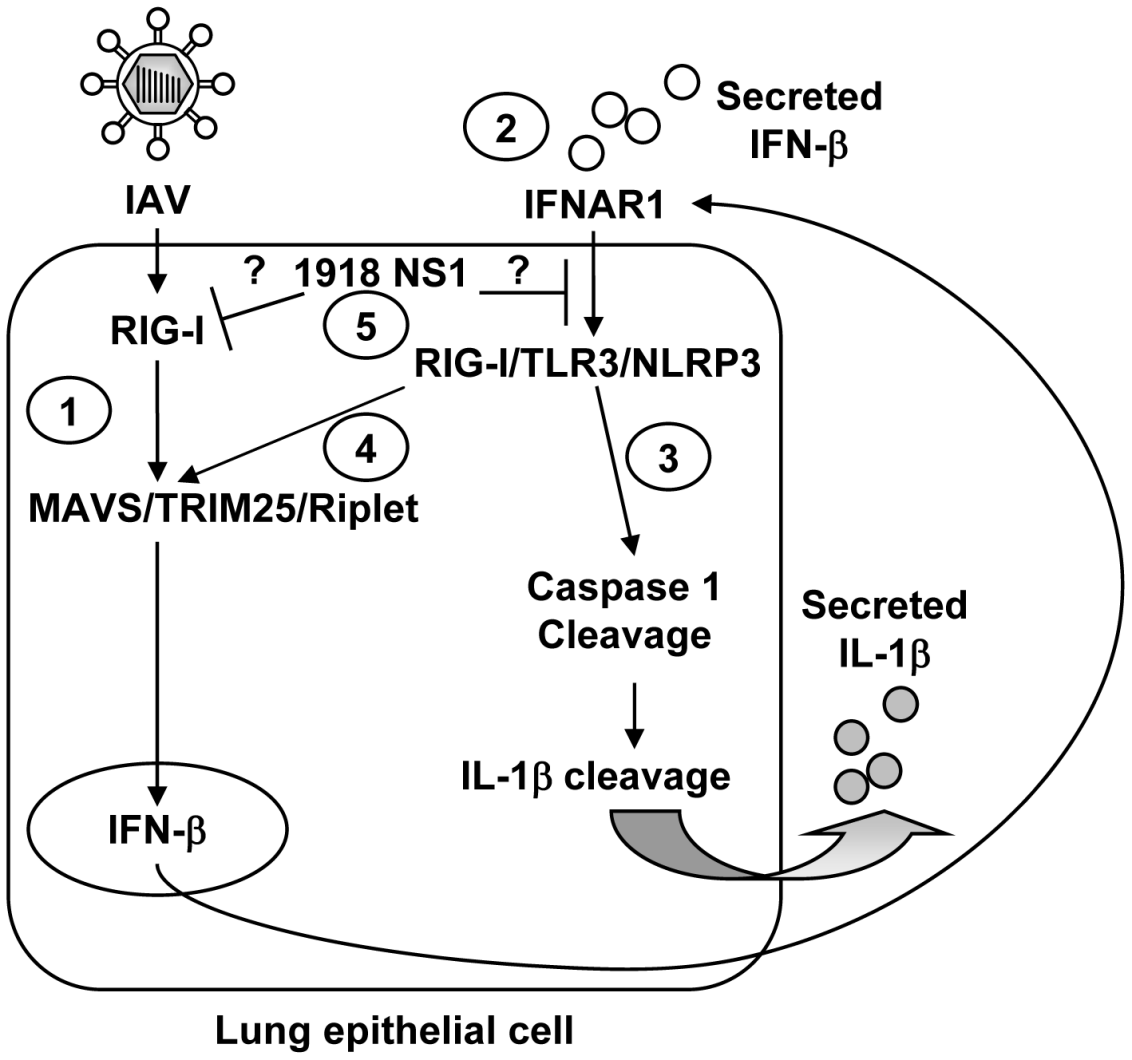
Proposed model of RIG-I as a pivotal regulator of the inflammasome response against IAV in lung epithelial cells. 1) Upon IAV infection, RIG-I senses viral genomic RNA and triggers a type I interferon response through a MAVS/TRIM25/Riplet-dependent pathway. 2) Binding of type I interferon to IFNAR1 initiates a positive feedback loop that stimulates RIG-I/TLR3/NLRP3 expression. 3) This upregulation is necessary for RIG-I- and NLRP3-inflammasome assembly, cleavage of caspase 1 and, ultimately, mature IL-1β secretion. 4) RIG-I upregulation stimulates type I IFN and constitutes an additional positive feedback loop. 5) 1918 NS1 could form more prominent complexes with RIG-I than USSR NS1, and/or target other cellular component(s) involved in type I IFN signaling, to result in a stronger inhibition of RIG-I-dependent type I interferon response by NS1 from the highly virulent pandemic 1918 influenza strain. 1918 NS1 protein-mediated inhibition of the type I IFN response consequently decreases IL-1β secretion.

Several lines of evidence support our conclusion that RIG-I is a pivotal inflammasome activator in IAV-infected human lung epithelial cells. First, although there was significant inhibition in TLR3 and NLRP3 knockdown cells, knockdown of RIG-I had the most dramatic effect on caspase 1 activation and IL-1β secretion, independent of the donor and of the viral strain used. Second, inhibition of the RIG-I signaling partners MAVS, TRIM25, and Riplet also resulted in significant inhibition of caspase 1 activation and IL-1β secretion. Third, WT RIG-I overexpression resulted in a significant upregulation of IL-1β secretion in response to IAV infection in reconstituted inflammasome experiments. In contrast, the S183I loss-of-function variant of RIG-I [25] resulted in inhibition of IL-1β secretion. Fourth, TLR3 and NLRP3 upregulation in IAV-infected NHBE cells is RIG-I dependent, as demonstrated both by RIG-I knockdown experiments and RIG-I overexpression assays. Thus, RIG-I operates upstream of TLR3- and NLRP3-dependent inflammasome activation. Fifth, stimulation of RIG-I is the major inducer of type I IFN responses in lung epithelial cells, as described here and previously [4], [5]; also type I IFN precedes and induces the IL-1β response and caspase 1 activation, as demonstrated by knockdown of IFN-β and IFNAR1 expression. Sixth, the functional demonstration that RIG-I directly activates the inflammasome in addition to its effect on the type I IFN response was provided by treatment of primary cells with IFN-β, which partially rescued the knockdown effect of RIG-I on the IL-1β response and cleaved caspase 1 secretion. These results mirror our observation with IFNAR1 and IFN-β knockdowns and support our hypothesis that RIG-I is critical for IFN-β-dependent IL-1β response, as IFN-β significantly increased inflammasome activation. It also supports the functional role of RIG-I *per se* in inflammasome activation, as IFN-β had only a partial rescue effect. Moreover, we observed that, in primary human lung epithelial cells, RIG-I directly interacted with caspase 1 and that IAV infection induces RIG-I binding to ASC, as shown in the context of VSV in a human cell line [29]. Thus, our data demonstrate that RIG-I directly promotes caspase 1 inflammasome activation in IAV-infected primary human lung epithelial cells and amplifies the IL-1β response through a type I IFN positive feed-back loop as shown in Figure 7.

To further validate the cross-talk between RIG-I, type I IFN, and IL-1β during IAV infection, we investigated the impact of influenza NS1 variants with a known effect on virulence and on the type I IFN response in ferrets [20]. It is of note that the virus carrying NS1 from the highly pathogenic 1918 influenza strain significantly inhibited the IL-1β response in ferrets and in human primary lung epithelial cells, as compared to NS1 from a seasonal IAV with moderate virulence [20], [30]. At least in primary lung epithelial cells, the 1918 NS1 also inhibited both type I IFN and RIG-I upregulation in response to IAV infection, suggesting that its effect on IFN induction contributes to inhibition of the IL-1β response. Furthermore, at a low MOI 1918 NS1 and USSR NS1 are similarly expressed in primary lung epithelial cells, but 1918 NS1 forms more prominent complexes with RIG-I than USSR NS1. This suggests that 1918 NS1 could induce a stronger inhibition of RIG-I activity than USSR NS1, which explains its inhibitory effect on the type I IFN and IL-1β responses. At a high MOI, 1918 NS1 was more abundant than USSR NS1, which may also contribute to its increased inhibitory effect. NS1 has been shown to inhibit type I IFN secretion by interacting with TRIM25 [18], an ubiquitin ligase critical for RIG-I function; as we show here, TRIM25 is essential for IL-1β secretion. However, the mechanism underlying the differences in inhibition observed for NS1 from the 1918 and USSR viral strains remains unclear, since the previously identified TRIM25-inhibiting residues E96, E97, R38, and K41 are conserved [18], [20]. In addition, NS1 is a multi-functional protein that interferes with the type I IFN response at several levels [2], [18], [20]–[23]. Similar to the regulation of RIG-I and type I IFN expression that we observed, macaques infected with the reconstructed 1918 pandemic virus showed considerably lower induction of these genes at day 3 post-infection in comparison with macaques infected with seasonal H1N1 [31]. Thus, we cannot exclude that the inhibitory effect associated with the 1918 NS1 protein could be, at least in part, due to the impact of NS1 on other cellular components involved in type I IFN signaling [22], [23]. Further studies involving NS1 site directed mutagenesis and reassortant viruses are necessary to determine whether the 1918 NS1 inhibits TRIM25-dependent RIG-I activation or other cellular components to limit antiviral and inflammasome responses, and which amino acid residues are involved in this function. Altogether, our data on 1918 NS1 raise new questions about the involved mechanism(s), but also demonstrate for the first time the impact of this negative regulator of type I IFN signaling on the lung epithelial cells IL-1β response (Figure 7). Furthermore, the data support our demonstration of a positive effect of type I IFN on the IL-1β response of lung epithelial cells with an approach using modified virus complementary to the specific targeting of host proteins with siRNA.

The influenza virus M2 protein triggers NLRP3-dependent, but likely RIG-I–independent, inflammasome activation in macrophages [11]. Conversely, in lung epithelial cells, NS1 inhibits RIG-I–dependent inflammasomes activation. These results underscore the complexity and specificity of virus interactions with different types of cells during pathogenesis. This suggests that the involvement of different PRRs in inflammasome activation in different cell types and their partial redundancy may play an important role in counteracting viral immune interference, for instance, through NS1 in lung epithelial cells. In line with this idea, 1918 NS1 strongly inhibited IL-1β secretion in lung epithelial cells, which is primarily RIG-I dependent, but did not affect the IL-1β response in macrophages, which is NLRP3 dependent (data not shown). One could hypothesize that the RIG-I–independent inflammasome response of macrophages confers resistance to viruses with an NS1 similar to 1918 NS1, which inhibits the IL-1β response in lung epithelial cells, but not in cells of myeloid origin.

In myeloid cells and in a mouse model of *Candida albicans* infection, the presence of type I IFNs inhibits the IL-1β response regulated by the activation of the NLRP3 inflammasome and increases mouse susceptibility to *C. albicans* infection [12]. These results are consistent with reported type I IFN anti-inflammatory effects, which could be due to inhibition of IL-1β production in some autoimmune diseases [32], [33]. Our results indicate an entirely different paradigm and suggest that conversely such treatment would be deleterious for diseases implicating the lung epithelium, including COPD or asthma [34], [35]. However, type I IFN is required for activation of the inflammasome in macrophages in response to cytosolic *Francisella novicida* and *Listeria monocytogenes*, but not vacuole-localized *Salmonella typhimurium* [36]. The type I IFN response to these cytosolic bacteria induces inflammasome-mediated cell death in macrophages [36]. Thus, the action of type I IFNs on IL-1β may be dependent on cell type and on the pathogen. Further IAV infection experiments in different cell types, including peripheral blood mononuclear cells and dendritic cells, will be important in determining whether the RIG-I–mediated type I IFN production that we observed is part of the general host response to IAV, or whether it is specific to lung epithelial cells. It is conceivable that the differential regulation of IL-1β by type I IFNs in non-immune versus immune cells reflects the different functions of type I IFNs, which are both antiviral and regulatory during the early infection of lung epithelial cells. At this time-point, it is important both to control virus growth rapidly and to produce sufficient IL-1β to promote cell recruitment [3], [27], [28]. However, at the point at which the macrophages become infected, the immunoregulatory role of type I IFNs may become predominant, to prevent potentially deleterious overproduction of IL-1β by macrophages or dendritic cells. Further studies of the cross-regulation of type I IFN and inflammasome responses are required to determine and contrast the beneficial and deleterious roles of type I IFN in inflammasome activation for virus control and clearance from the host.

Additional experiments are also required to examine whether our results can be generalized to the native epithelium. One approach would be to use cells cultured in an air-liquid interface, which produces polarized pseudostratified mucociliary cells resembling natural airway epithelial cells. Under these culture conditions, well-differentiated epithelial cells produce a reduced inflammatory response and are more resistant to rhinovirus infection than undifferentiated cells [37]. The enhanced resistance to IAV infection of these differentiated cells could contribute to the control of the inflammatory response of epithelial cells. Furthermore, it has been shown that cytokine secretion in response to IAV infection is polarized towards the apical and basolateral membranes, whereas IFNAR expression is restricted to the basolateral membrane [9], [38]. The localization of IFNAR limits its stimulation by type I IFN to the basolateral side, which could further reduce the extent of the positive-feed back loop acting on IL-1β secretion in differentiated epithelial cells. However, yet another scenario is conceivable: *in vivo*, epithelial cells are in close proximity to dendritic cells, which usually reside near the basolateral membrane. In response to virus infection, dendritic cells produce large amounts of type I IFNs, which could act in a paracrine manner to efficiently amplify the production of IL-1β by the airway epithelium. Of note, however, upon IAV infection IFNAR-deficient or STAT1-deficient differentiated mouse epithelial cells show a significant reduction of IL-1β production compared to wild-type controls [9]. Thus, although the extent of the IL-1β response in differentiated epithelial cells infected with IAV cells remains to be established *in vivo*, the available data indicate that, at least in mouse cells, IL-1β and type I IFN production are positively correlated in differentiated epithelial cells, in a similar fashion to what we have observed in undifferentiated primary human epithelial cells.

Our results showing that type I IFN controls inflammasome activation and production of bioactive IL-1β add to our knowledge of how airway epithelial cells respond to infection and regulate lung innate and adaptive immunity [8], [9], [39]. Whereas a large body of evidence supports the critical role of IFNAR/STAT1 axis for both lung epithelial immunity as well as the subsequent adaptive immunity [8], [9], the mechanisms that mediate these responses are yet not entirely clear. IL-1β is central in the communication between epithelial cells and monocytes during the initiation of inflammation and development of the adaptive response. However, IL-1β can also act in an autocrine manner on epithelial cells infected with rhinovirus to elicit secretion of CXCL8, a potent neutrophil attractant, and control of viral replication [40]. In addition to long-range signals, epithelial cell IL-1β may have a role in regulating the local homeostasis of the lung epithelium. Epithelial cells express and secrete a battery of immune modulators the role of which is to limit innate inflammation in this environmentally exposed site, including surfactant proteins A and D and mucin 1, which suppress the activity of alveolar macrophages [41]. Furthermore, alveolar macrophages display a unique response to PRR agonists, characterized by a strong inflammatory response but lacking autocrine/paracrine IFN-β secretion and STAT-1 activation [42]. Thus, it is tempting to speculate that lung epithelial cells, which display a distinctive RIG-I–mediated type I IFN antiviral responses and inflammasome activation, actively participate in the orchestration of lung immune response by providing simultaneously IFN-β and IL-1β paracrine stimulation to alveolar macrophages. Such epithelial responses to infection could provide rapid and robust local immunity to rapidly curb viral load, stimulate immune cells neighboring the affected site while limiting inflammation to the local environment. To clarify these mechanisms conditional or cell type-specific knock-out mice of IL-1β will be required.

The targeting of RIG-I–like receptors (RLR) with specific agonists has been proposed as a prophylaxis and treatment for IAV, as it may increase antiviral innate and adaptive immunity, leading to more rapid elimination of the virus [43], [44]. Our results suggest that these therapeutic strategies will not only initiate a powerful antiviral response, but also trigger a potent IL-1β response via RIG-I. Further studies of the tissue specificity of the RIG-I–dependent inflammasome may be useful to determine if other epithelial tissues share the same specificity that we observed in respiratory epithelial cells. This will improve our understanding of whether RLR agonists may be a useful addition to our antiviral therapeutic arsenal, and if they may be instrumental in the next generation of vaccine adjuvants.

## Materials and Methods

### Ethics statement

This study was carried in strict accordance with the recommendations of the Guide for the Care and Use of Laboratory Animals of the INRS-Institut Armand-Frappier and the Canadian Council on Animal Care (protocol # 0911-02). In accordance with current US regulations governing tissue banking in the Code of Federal Regulations, 21 CFR Part 1271, human tissue was acquired by Lonza through donor programs that perform tissue recovery and donor informed consent following processes approved by an Institutional Review Board.

### IAV infection in ferrets

Male ferrets (*Mustela putorius furo*) were infected intranasally as described previously [20].

### Viruses and primary NHBE cells

The H1N1 viruses, PR8 and USSR, were described previously [30]. NS PR8, NS1 1918, and WT recombinant USSR (rUSSR) viruses were generated from cloned cDNA as described previously [20]. All viruses were propagated in MDCK cells (ATCC CCL-34) in F-12K medium with 2 µg/ml trypsin (TRTPCK Worthington). IAV titers were obtained by standard plaque assay on confluent monolayers of MDCK cells and expressed as pfu/ml. Primary NHBE cells (CC-2541, Lonza) from five male donors (three of Caucasian origin [111011, 75008, 4F1289J] and two of African-American ancestry [118008, 7F4120]; see Table S1) were grown in complete Clonetics BEGM BulletKit (Lonza) according to the manufacturer's instructions.

### IAV infection and reagent stimulation of human primary lung epithelial cells

NHBE cells were seeded at 8,750 cells/well 9 days before infection. All cells were seeded in 24-well plates and washed twice with 200 µl/well OptiMEM medium (Invitrogen) before infection with IAV or stimulation with poly(I:C) (1 or 5 µg/ml, SIGMA), poly(A:U) (100 µg/ml, InvivoGen), or nigericin (10 µM, InvivoGen) diluted in OptiMEM (200 µl/well). In IFN-β pretreatment experiments, NHBE cells were pretreated with human IFN-β (8–1,000 IU/ml, PBL InterferonSource) before challenge with poly(I:C) or nigericin.In IFN-β treatment experiments of siRNA transfected NHBE cells, cells were mock treated or infected with IAV (MOI 1) 48 h after siRNA transfection (as described below). 8 h later post-infection cells were treated for 16 h with 200 IU/ml of human IFN-β.

### siRNA-mediated gene silencing

NHBE cells were seeded as described above 7 days before transfection with siRNAs (10 nM) in 3 µl/well of HiPerfect transfection reagent (Qiagen) according to the manufacturer's instructions. Nontargeting siRNAs were used as controls in all experiments. Target sequences of the siRNAs are provided in Table S5. Cells were mock treated or infected with IAV (MOI 1) 48 h after siRNA transfection.

### Study of endogenous interactions in NHBE primary lung epithelial cells

NHBE cells were mock treated or infected with either USSR or rUSSR-NS1 1918 for 18 h. Cells were directly disrupted into the well by adding 1% Triton X-100, 10 mM of dithiothreitol, and a protease inhibitor cocktail. Samples were further processed as described previously [25], and coimmunoprecipitation was performed in a 600 µl volume on 2400 µg of cell lysate with 50 µl of protein G sepharose (BioVision) and 10 µg of either anti-RIG-I (ALX-804-849; Enzo Life Science), anti-ASC (ALX-210-905; Enzo Life Science), anti-procaspase 1 (#2225; Cell Signaling Technology) or the control IgG (anti-FLAG, M2, SIGMA) antibody; protein complexes were analyzed by immunoblotting as described previously [25] with antibodies against RIG-I, ASC or pro-caspase 1.

### Quantitative RT-PCR, LDH assay, percentage of living cells, immunoblots, ELISA and IL-1β reporter assay

These procedures are detailed in the supplemental experimental procedures.

### Statistical analysis

Unless otherwise stated, data are presented as mean ± SEM of triplicate or quadruplicate samples from at least three independent experiments. Statistical differences were tested using one-way ANOVA followed by Fisher's test, with a threshold of *p*<0.05 in all Figures. Correlation analyses were performed using the Pearson's Correlation test, with a threshold of *p*<0.05. Probability indicated by asterisks: *p<0.05, **p<0.01, and ***p<0.001. In experiments with siRNA-transfected cells, mock-treated or IAV-infected cells transfected with specific siRNAs were respectively compared to their control siRNA-transfected cells counterparts.

## Supporting Information

Figure S1 **As with the response to USSR, RIG-I–dependent caspase 1 activation is pivotal for the IL-1β response to PR8 IAV.** For (A) to (D) NHBE cells (n = 3 donors) were first transfected with siRNA (either a control or one targeting either RIG-I, TLR3 or NLRP3) and then either mock-treated (Mock) or infected with PR8 (MOI 1, +PR8) for 18 h. (A) Secreted IL-1β and (B) active cleaved caspase 1 p20 from the cell-free supernatant. (C) IL-1β secretion normalized with respect to total (intracellular and secreted) IL-1β production and expressed as a percentage. (D) Percentage of IL-1β production with respect to cells transfected with control siRNA and infected with PR8. (A–D) Data are presented as the mean ± SEM of quadruplicates. *p<0.05, **p<0.01, and ***p<0.001, respectively comparing mock-treated or IAV-infected cells transfected with a specific siRNA to their control siRNA-transfected counterparts.
(PDF)

Figure S2 **Differential IL-1β secretion is not due to the stimulation of pro-IL-1β protein expression by control siRNA transfection, to a significant pro-caspase 1 protein downregulation nor to the inhibition of cell growth in targeted knockdowns.** (A) Immunoblot detection of pro-IL-1β and β-actin in NHBE cells either mock-treated (−) or infected with USSR (+) following transfection with control siRNA or without siRNA transfection (w/o siRNA). (B) Immunoblot detection of pro-caspase 1 and β-actin in knockdown cells from two different NHBE donors (111011 and 4F1289J). The cells were first transfected with either a control or specific siRNA (targeting either RIG-I, TLR3, NLRP3, MAVS, TRIM25 or Riplet) and then either mock-treated (−) or infected with USSR (+). (C) Total LDH activity in knockdown cell lysates and supernatants from 3 different NHBE donors. *p<0.05, **p<0.01, and ***p<0.001, respectively comparing mock-treated or USSR-infected cells transfected with a specific siRNA to their control siRNA-transfected counterparts.
(PDF)

Figure S3 **RIG-I overexpression increases TLR3 and NLRP3 expression in both HEK 293T and NHBE cells.** NHBE (A, C and E) or HEK 293T (B, D and F) cells were transfected with an empty vector (control), or a FLAG-WT RIG-I expression vector (RIG-I). The cells were then either mock treated or infected with USSR (NHBE) or PR8 (HEK 293T). NLRP3 (A, NHBE cells; B, HEK 293T cells) and TLR3 (C, NHBE cells; D, HEK 293T cells) mRNA expression in transfected cells 13 h after mock (−) or IAV infection (+IAV). Results are presented as mean fold increase ± SEM relative to mock treated cells transfected with control vectors, normalized to β-actin levels. Results are presented as pool of three independent experiments. (E and F) Immunoblots of overexpressed FLAG-RIG-I (RIG-I) and β-actin in transfected NHBE cells (E) and HEK 293T cells (F) from the same experiments. *p<0.05, **p<0.01, ***p<0.001, respectively comparing mock-treated or IAV-infected cells transfected with RIG-I to their empty vector-transfected (control) counterparts.
(PDF)

Figure S4 **IFN-β expression in NHBE cells controls virus replication, but not caspase 1 expression.** NHBE cells were transfected with control or IFN-β-, IFNAR1-, or RIG-I–targeting siRNA followed by mock or USSR (+IAV) infection for 18 h. (A) Ratio of IFN-β protein signals on immunoblots normalized with respect to the β-actin signal. (B) Caspase 1 mRNA expression. Data are presented as mean ± SEM mRNA expression, derived from eight qRT-PCRs, spread over four independent experiments and using cells from four donors, normalized with respect to β-actin level. Results are expressed as fold-increase relative to control siRNA transfected cells treated with mock. (C) Ratio of NS1 protein signals on immunoblots normalized with respect to the β-actin signal. (A–C) *p<0.05, **p<0.01, and ***p<0.001, respectively comparing mock-treated or IAV-infected cells transfected with specific siRNA to their control siRNA-transfected counterparts.
(PDF)

Figure S5 **1918 NS1 increases viral RNA in human NCI-H292 cells at latter time points but not at early time points post-infection.** Kinetics of rUSSR-NS1 1918 and WT rUSSR M2 RNA expression in NCI-H292 cells. Total RNA was isolated from NCI-H292 cells infected with mock, WT rUSSR or rUSSR-NS1 1918 (MOI 0.5) for various times. IAV M2 RNA expression was examined by qRT-PCR. Gene expression was normalized to that of β-actin. Results are mean ± SEM of quadruplicate qRT-PCR presented as fold-increase relative to WT rUSSR 3 h post-infection. **p<0.01 and ***p<0.001 rUSSR-NS1 1918-infected compared with WT rUSSR–infected cells.
(PDF)

Protocols S1 **Supplemental experimental procedures.**
(RTF)

Table S1 **NHBE donors.**
(RTF)

Table S2 **Total IL-1β (intracellular and seåcreted IL-1β) in siRNA- transfected NHBE cells (data integrated in** **Figure 3B** **).**
(RTF)

Table S3 **Secreted IL-1β in the supernatant of siRNA-transfected NHBE cells integrated in** **Figure 3B** **.**
(RTF)

Table S4 **qRT-PCR primer sequences.**
(RTF)

Table S5 **Target sequence of short interfering RNA (siRNA)**
(RTF)

References S1 **Supplemental data references.**
(RTF)
