# Supplementary figures and images for: Type I IFN Triggers RIG-I/TLR3/NLRP3-dependent Inflammasome Activation in Influenza A Virus Infected Cells
Source: PLoS Pathog. 2013 Apr 11;9(4):e1003256. doi: 10.1371/journal.ppat.1003256 (PMC3623797; doi:10.1371/journal.ppat.1003256)

Fig. S1 Pothlichet et al.,

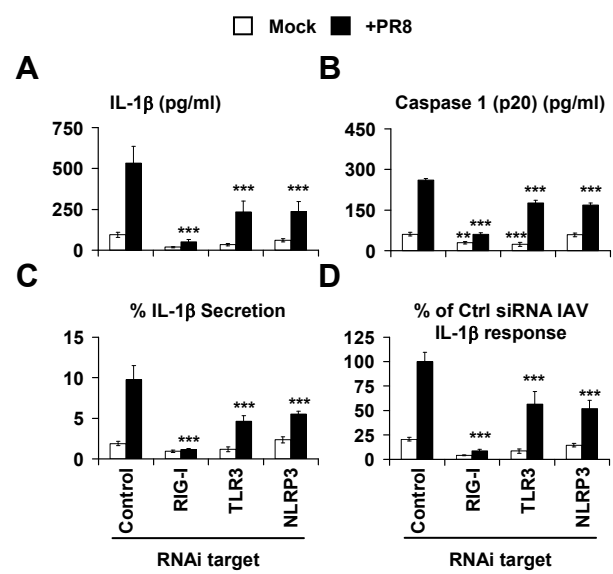

Supplement: Figure S1 — As with the response to USSR, RIG-I–dependent caspase 1 activation is pivotal for the IL-1β response to PR8 IAV. For (A) to (D) NHBE cells (n = 3 donors) were first transfected with siRNA (either a control or one targeting either RIG-I, TLR3 or NLRP3) and then either mock-treated (Mock) or infected with PR8 (MOI 1, +PR8) for 18 h. (A) Secreted IL-1β and (B) active cleaved caspase 1 p20 from the cell-free supernatant. (C) IL-1β secretion normalized with respect to total (intracellular and secreted) IL-1β production and expressed as a percentage. (D) Percentage of IL-1β production with respect to cells transfected with control siRNA and infected with PR8. (A–D) Data are presented as the mean ± SEM of quadruplicates. *p<0.05, **p<0.01, and ***p<0.001, respectively comparing mock-treated or IAV-infected cells transfected with a specific siRNA to their control siRNA-transfected counterparts. (PDF) [file ppat.1003256.s001.pdf]

Fig. S2 Pothlichet et al.,

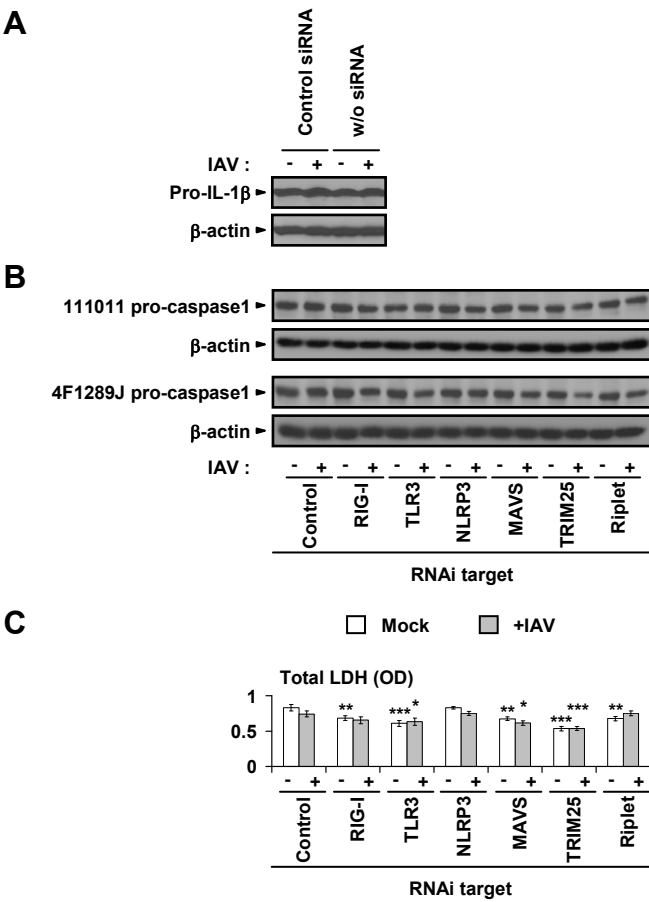

Supplement: Figure S2 — Differential IL-1β secretion is not due to the stimulation of pro-IL-1β protein expression by control siRNA transfection, to a significant pro-caspase 1 protein downregulation nor to the inhibition of cell growth in targeted knockdowns. (A) Immunoblot detection of pro-IL-1β and β-actin in NHBE cells either mock-treated (−) or infected with USSR (+) following transfection with control siRNA or without siRNA transfection (w/o siRNA). (B) Immunoblot detection of pro-caspase 1 and β-actin in knockdown cells from two different NHBE donors (111011 and 4F1289J). The cells were first transfected with either a control or specific siRNA (targeting either RIG-I, TLR3, NLRP3, MAVS, TRIM25 or Riplet) and then either mock-treated (−) or infected with USSR (+). (C) Total LDH activity in knockdown cell lysates and supernatants from 3 different NHBE donors. *p<0.05, **p<0.01, and ***p<0.001, respectively comparing mock-treated or USSR-infected cells transfected with a specific siRNA to their control siRNA-transfected counterparts. (PDF) [file ppat.1003256.s002.pdf]

Fig. S3 Pothlichet et al.,

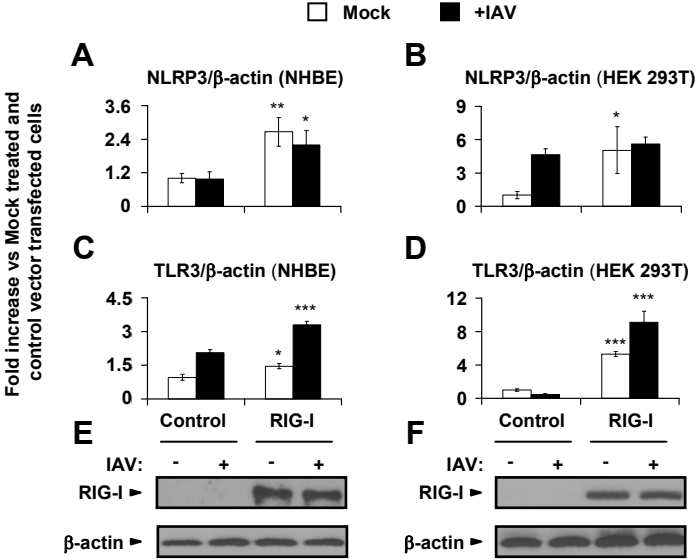

Supplement: Figure S3 — RIG-I overexpression increases TLR3 and NLRP3 expression in both HEK 293T and NHBE cells. NHBE (A, C and E) or HEK 293T (B, D and F) cells were transfected with an empty vector (control), or a FLAG-WT RIG-I expression vector (RIG-I). The cells were then either mock treated or infected with USSR (NHBE) or PR8 (HEK 293T). NLRP3 (A, NHBE cells; B, HEK 293T cells) and TLR3 (C, NHBE cells; D, HEK 293T cells) mRNA expression in transfected cells 13 h after mock (−) or IAV infection (+IAV). Results are presented as mean fold increase ± SEM relative to mock treated cells transfected with control vectors, normalized to β-actin levels. Results are presented as pool of three independent experiments. (E and F) Immunoblots of overexpressed FLAG-RIG-I (RIG-I) and β-actin in transfected NHBE cells (E) and HEK 293T cells (F) from the same experiments. *p<0.05, **p<0.01, ***p<0.001, respectively comparing mock-treated or IAV-infected cells transfected with RIG-I to their empty vector-transfected (control) counterparts. (PDF) [file ppat.1003256.s003.pdf]

Fig. S4 Pothlichet et al.,

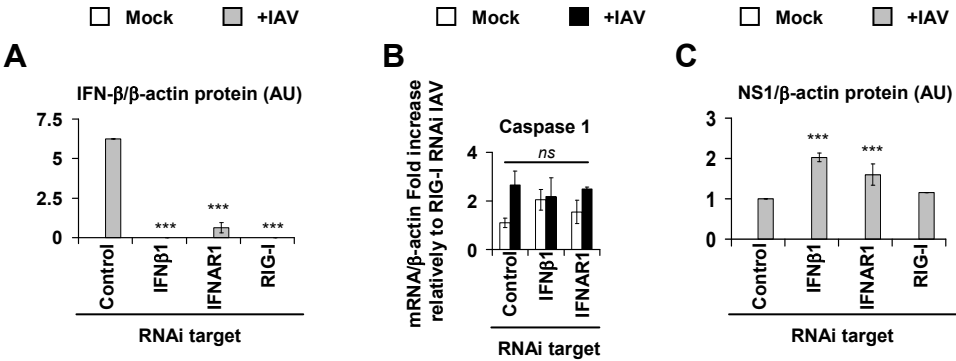

Supplement: Figure S4 — IFN-β expression in NHBE cells controls virus replication, but not caspase 1 expression. NHBE cells were transfected with control or IFN-β-, IFNAR1-, or RIG-I–targeting siRNA followed by mock or USSR (+IAV) infection for 18 h. (A) Ratio of IFN-β protein signals on immunoblots normalized with respect to the β-actin signal. (B) Caspase 1 mRNA expression. Data are presented as mean ± SEM mRNA expression, derived from eight qRT-PCRs, spread over four independent experiments and using cells from four donors, normalized with respect to β-actin level. Results are expressed as fold-increase relative to control siRNA transfected cells treated with mock. (C) Ratio of NS1 protein signals on immunoblots normalized with respect to the β-actin signal. (A–C) *p<0.05, **p<0.01, and ***p<0.001, respectively comparing mock-treated or IAV-infected cells transfected with specific siRNA to their control siRNA-transfected counterparts. (PDF) [file ppat.1003256.s004.pdf]

Fig. S5 Pothlichet et al.,

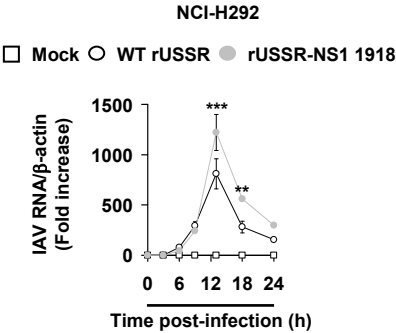

Supplement: Figure S5 — 1918 NS1 increases viral RNA in human NCI-H292 cells at latter time points but not at early time points post-infection. Kinetics of rUSSR-NS1 1918 and WT rUSSR M2 RNA expression in NCI-H292 cells. Total RNA was isolated from NCI-H292 cells infected with mock, WT rUSSR or rUSSR-NS1 1918 (MOI 0.5) for various times. IAV M2 RNA expression was examined by qRT-PCR. Gene expression was normalized to that of β-actin. Results are mean ± SEM of quadruplicate qRT-PCR presented as fold-increase relative to WT rUSSR 3 h post-infection. **p<0.01 and ***p<0.001 rUSSR-NS1 1918-infected compared with WT rUSSR–infected cells. (PDF) [file ppat.1003256.s005.pdf]
