# Supplementary material for: Type I IFN Triggers RIG-I/TLR3/NLRP3-dependent Inflammasome Activation in Influenza A Virus Infected Cells
Source: PLoS Pathog. 2013 Apr 11;9(4):e1003256. doi: 10.1371/journal.ppat.1003256 (PMC3623797; doi:10.1371/journal.ppat.1003256)
Supplement: Protocols S1 — Supplemental experimental procedures. (RTF) [file ppat.1003256.s006.rtf]

Supplemental experimental proceduresQuantitative real-time PCR (qRT-PCR) analysis	cDNA was obtained from 1 μ of total RNA, as follows: total RNA was extracted using the RNeasy Kit (Qiagen), treated with DNase I (Roche), and finally reverse transcribed with MMLV reverse transcriptase (Invitrogen) according to the manufacturer’s instructions. IAV M2 RNA levels, as well as βactin, RIG-I, TLR3, NLRP3, Riplet, IFN-β IFNAR1, ASC, and caspase 1 mRNA levels were quantified using the SYBR Green PCR master mix, according to the manufacturer’s instructions. Primer sequences and procedure details are provided in Table S4. Duplicate cycle threshold (CT) values were analyzed with the comparative CT (ΔΔT) method (Applied Biosystems). The relative amount of mRNA (2−ΔΔT) was obtained by normalizing to the endogenous βactin reference level in all experiments. Ferret IL-1βand IFN-βexpression in nasal washes was quantified by qRT-PCR with specific primers for those genes (see sequences in Table S4), which were designed based on ferret sequences (NCBI accession numbers: IFN-β EF368208 and IL-1β AB301555.1). RNA from nasal wash cells was obtained from a previous study and qRT-PCR was performed as described previously [1]. Briefly, nasal washes were collected daily for the first 3 days post-infection, as described in reference [1]. To do so, 500 µl of PBS was instilled in one nostril and the expectorate was collected in a 50 ml centrifuge tube. Immediately after collection, 140 μ of nasal wash fluid was mixed with 560 μ of AVL buffer (QIAamp Viral RNA Mini kit; Qiagen). RNA was extracted according to the manufacturer’s instructions and qRT-PCR was performed as described previously [1]. The number of RNA copies was determined according to the following formula: number of copies = amount of RNA (ng) × 6.022 × 1023/mRNA length (base) × 109 × 340.Lactate dehydrogenase (LDH) assay to determine the percentage of living cells 	The percentage of living cells was determined with the LDH assay kit (Cytotoxicity Detection KitPLUS, Roche), based on the quantification of LDH activity in the cytosol of dying cells. Among the cells stimulated in 200 µl of medium, 100 µl were collected to quantify LDH activity. Cells were then disrupted in the 100 µl of remaining supernatant using 20 µl of lysis buffer (Roche) to quantify total (intracellular plus extracellular) LDH activity. Percentage of living cells = 100 −LDH activity released in the supernatant/total LDH activity.Immunoblotting	To study gene silencing effects at the protein level, NHBE cells cultured in 24-well plates were disrupted for 30 min at 4°C using 150 µl of a lysis buffer, which contains 50 mM Tris-HCl (pH 7.6), 1 mM EDTA, 150 mM NaCl, 1% Triton X-100, 10% glycerol, 1% dithiothreitol, and a protease inhibitor cocktail. Cytosolic proteins were recovered from the supernatant after a 15-minute centrifugation (15,000 × g, 4°C). Protein concentrations were determined using BCA reagent (Pierce). Protein samples were treated as previously described [2], then electrotransferred onto a nitrocellulose membrane and probed using an antibody specific for RIG-I (from mouse, 1:2,000, ALX-804-849; Enzo Life Science; from rabbit, 1:1000, #4520, Cell Signaling Technology), MAVS (1:2,000, ALX-210-929; Enzo Life Science), TRIM25 (1:15,000, #610570; BD Transduction Laboratories), ASC (1:1,000, ALX-210-905; Enzo Life Science), NS1 (1:200, sc-130563; Santa Cruz Biotechnology), βactin (1:10,000, #A 2228; Sigma-Aldrich), cleaved (p17) IL-1β(1:1,000, #2021; Cell Signaling Technology), pro-IL-1β(1:250, MAB201, R&D), pro-caspase 1 (1:1,000, #2225; Cell Signaling Technology), and cleaved caspase-3 (1:1,000, #9664; Cell Signaling Technology). Bound antibodies were detected using the SuperSignal West Femto Maximum Sensitivity Substrate (Thermo Scientific) according to the manufacturer’s instructions.ELISA	Protein concentrations in cell-free supernatant were determined using the Quantikine ELISA kit for human cleaved p20 caspase 1 (R&D Systems), the Duoset ELISA kit (R&D Systems) for human cytokine IL-1β(DY201) or the VeriKine human IFN-βELISA kit (41410, PBL interferon source). Percentage of control siRNA IAV IL-1βresponse = IL-1βresponse in knockdown cells/control siRNA IL-1βresponse in IAV-infected cells × 100. Total IL-1βrepresents intracellular (in cell lysates) and secreted (in supernatants) IL-1βproduction in samples prepared for total LDH activity quantification, as described above in the LDH assay procedure. Intracellular IL-1β= total −secreted IL-1βamount. Percentage of IL-1βsecretion = secreted IL-1βtotal IL-1βin the same sample × 100.IL-1βreporter assay	HEK 293T cells seeded into 6-well plates were transiently cotransfected using Fugene 6 (Roche), as described previously [3], with 2 µg of a control plasmid (pEFBOS(+)) or pEFBOS vectors encoding either Flag-tagged (N-ter) WT RIG-I or S183I RIG-I, as described previously [3], together with 250 ng of pCDNA3.1D-pro-hIL-1βFlag (Cter), 31.25 ng of pCDNA3.1D-pro-caspase 1-V5-His, 8 ng of pCDNA3-MycASC and 100 ng of pRSV-βgal (to check transfection efficiency). Cells were either mock treated or infected with PR8 virus (MOI 1) 24h after transfection. Cell-free supernatants were collected 16h p.i. for IL-1βanalysis; cells were directly disrupted inside the well by adding 1% Triton X-100, 1% dithiothreitol, and a protease inhibitor cocktail. IL-1βconcentration was determined by ELISA; βgalactosidase activity was measured by mixing 20 µl of cell lysate with 50 µl of buffer containing 1.33 mg/ml of ONPG substrate, 100 mM of 2-mercaptoethanol, 2 mM MgCl2, and 200 mM of Na2HPO4 (pH 7.3). Activity was quantified at 450 nm with a plate reader. IL-1βresponse is presented after normalization to βgalactosidase activity.RIG-I overexpression assays in HEK 293T cells and primary NHBE cells:	HEK293 T cells were seeded at 1.33x10e5 cells/well in 24-well plates the day before transfection. Cells were transfected using Fugene 6 (Roche) with 400ng of control plasmid (pEFBOS(+)) or pEFBOS vector encoding Flag-tagged (N-ter) WT RIG-I as described previously [3]. NHBE cells were seeded at 8,750 cells/well 7 days before transfection with 100ng of control plasmid or a WT RIG-I expression vector described above in 0.8µl/well of Enhancer and 5µl of Effectene transfection reagent (Qiagen, 301425) according to the manufacturer’s instructions. Cells were mock treated or infected with IAV (MOI 1; PR8 on 293T cells; USSR on NHBE cells) 34 h after transfection in 200 µl/well of OptiMEM medium. RNA and proteins were collected from 293T cells and NHBE cells 13h post-IAV infection.IAV infection of human NCI-H292 cells:	Human NCI-H292 lung epithelial cells (ATCC CRL-1848) were seeded at 2 × 10^5^ cells/well in 500 µl of F-12K medium (Multicell) supplemented with antibiotics and 10% fetal calf serum (Hyclone) 24 h before infection. Cells were seeded in 24-well plates and washed twice with 200 µl/well OptiMEM medium (Invitrogen) before infection with IAV diluted in OptiMEM (200 µl/well).References1. Meunier, I., and von Messling, V. (2011). NS1-mediated delay of type I interferon induction contributes to influenza A virulence in ferrets. J Gen Virol 92, 1635-1644.2. Pothlichet, J., Chignard, M., and Si-Tahar, M. (2008). Cutting edge: innate immune response triggered by influenza A virus is negatively regulated by SOCS1 and SOCS3 through a RIG-I/IFNAR1-dependent pathway. J Immunol 180, 2034-2038.3. Pothlichet, J., Burtey, A., Kubarenko, A.V., Caignard, G., Solhonne, B., Tangy, F., Ben-Ali, M., Quintana-Murci, L., Heinzmann, A., Chiche, J.D., et al. (2009). Study of human RIG-I polymorphisms identifies two variants with an opposite impact on the antiviral immune response. PLoS One 4, e758
