# Supplementary material for: Type I IFN Triggers RIG-I/TLR3/NLRP3-dependent Inflammasome Activation in Influenza A Virus Infected Cells
Source: PLoS Pathog. 2013 Apr 11;9(4):e1003256. doi: 10.1371/journal.ppat.1003256 (PMC3623797; doi:10.1371/journal.ppat.1003256)
Supplement: Table S1 — NHBE donors. (RTF) [file ppat.1003256.s007.rtf]

Table S1: NHBE donorsDonor#	Donor Age	Donor Ethnic Background	
75008	54 Y	Caucasian	
111011	67 Y	Caucasian	
4F1289J	24 Y	Caucasian	
118008	19 Y	African American	
7F4120	19 Y	African American	
