# Supplementary material for: Type I IFN Triggers RIG-I/TLR3/NLRP3-dependent Inflammasome Activation in Influenza A Virus Infected Cells
Source: PLoS Pathog. 2013 Apr 11;9(4):e1003256. doi: 10.1371/journal.ppat.1003256 (PMC3623797; doi:10.1371/journal.ppat.1003256)
Supplement: Table S2 — Total IL-1β (intracellular and seåcreted IL-1β) in siRNA- transfected NHBE cells (data integrated in Figure 3B ). (RTF) [file ppat.1003256.s008.rtf]

Table S2: Total IL-1β(intracellular and secreted IL-1β in siRNA-transfected NHBE cells (data integrated in Figure 3B)Total IL-1β(pg/ml)									
 	Donor	Control	RIG-I	TLR3	NLRP3	MAVS	TRIM25	RNF135	
Mock	4F1289J	5330	2651	3421	3689	2711	1155	2811	
 	 	5301	2361	2962	3330	3040	1263	3314	
 	 	5315	2035	2989	3339	2394	1432	3709	
 	 	5179	2488	3144	3824	3071	 	2534	
 	75008	4002	2570	3645	2110	2569	1424	4602	
 	 	6357	2374	3179	2317	2743	1154	4103	
 	 	5860	2086	3340	2722	2965	1323	4194	
 	 	4905	1617	3730	2453	3159	 	3910	
 	118008	4980	1401	3977	3754	4036	2796	4973	
 	 	5505	1491	3520	2885	3708	2803	5071	
 	 	5293	1784	3896	3103	4563	3406	5596	
 	 	5347	1950	4529	1788	4443	 	6139	
USSR	4F1289J	2580	2648	2406	1944	2418	1058	2922	
 	 	2522	2746	2556	1669	2369	1121	4211	
 	 	2254	2413	2268	1382	2071	891	3216	
 	 	2259	3115	2647	2686	1733	 	3500	
 	75008	3437	1410	2926	1907	3534	2783	4267	
 	 	2412	1584	3579	1951	2969	2537	3319	
 	 	1901	1103	2588	1709	3356	2597	4170	
 	 	 	1277	2905	2410	3285	 	4737	
 	118008	4024	3316	2449	1332	2138	1160	3414	
 	 	3114	2870	3656	1143	2212	850	3203	
 	 	2954	2565	3395	1079	2385	795	3708	
 	 	2500	1701	2940	1456	2516	 	3984	
PR8	4F1289J	5832	4338	4320	4956	 	 	 	
 	 	5591	4121	4540	4086	 	 	 	
 	 	5179	4058	3920	3887	 	 	 	
 	 	5453	4641	3993	4441	 	 	 	
 	75008	4858	2828	3375	3232	 	 	 	
 	 	5725	3274	3546	2996	 	 	 	
 	 	5592	3388	3433	2919	 	 	 	
 	 	5877	3963	3150	3646	 	 	 	
 	118008	5476	3148	4656	3778	 	 	 	
 	 	4748	2136	4225	2502	 	 	 	
 	 	5453	2442	4821	3862	 	 	 	
 	 	5278	2420	5259	4258	 	 		
