# Supplementary material for: Type I IFN Triggers RIG-I/TLR3/NLRP3-dependent Inflammasome Activation in Influenza A Virus Infected Cells
Source: PLoS Pathog. 2013 Apr 11;9(4):e1003256. doi: 10.1371/journal.ppat.1003256 (PMC3623797; doi:10.1371/journal.ppat.1003256)
Supplement: Table S3 — Secreted IL-1β in the supernatant of siRNA-transfected NHBE cells integrated in Figure 3B . (RTF) [file ppat.1003256.s009.rtf]

Table S3: Secreted IL-1βin the supernatant of siRNA-transfected NHBE cells integrated in Figure 3BSecreted IL-1â (pg/ml)									
 	Donor	Control	RIG-I	TLR3	NLRP3	MAVS	TRIM25	RNF135	
Mock	4F1289J	98	20	23	47	63	16	44	
 	 	87	9	11	50	56	9	33	
 	 	51	10	32	70	44	13	66	
 	 	91	22	11	43	 	 	47	
 	75008	115	43	65	105	100	48	184	
 	 	209	33	37	70	47	19	102	
 	 	156	27	59	92	48	25	117	
 	 	157	32	133	97	93	 	179	
 	118008	71	12	59	75	35	23	93	
 	 	58	7	18	34	31	14	71	
 	 	52	15	25	51	40	9	65	
 	 	58	9		35	65	 	96	
USSR	4F1289J	661	91	199	153	217	112	185	
 	 	652	97	266	117	286	110	175	
 	 	588	89	206	98	218	90	197	
 	 	 	133	307	 	225	 	249	
 	75008	1557	96	641	316	381	647	308	
 	 	934	112	494	230	353	371	222	
 	 	719	78	606	250	338	402	251	
 	 	 	51	704	419	360	 	301	
 	118008	598	91	161	127	160	118	217	
 	 	411	83	172	89	179	58	187	
 	 	329	92	175	87	173	78	270	
 	 	426	55	170	181	220	 	344	
PR8	4F1289J	535	66	304	357	 	 	 	
 	 	397	54	351	258	 	 	 	
 	 	438	50	210	195	 	 	 	
 	 	617	86	319	327	 	 	 	
 	75008	662	29	78	156	 	 	 	
 	 	644	26	83	148	 	 	 	
 	 	1163	46	71	142	 	 	 	
 	 	1126	68	78	228	 	 	 	
 	118008	398	45	127	139	 	 	 	
 	 	163	11	137	88	 	 	 	
 	 	197	12	270	211	 	 	 	
 	 	123	14	367	289	 	 	 	
