# Supplementary material for: Type I IFN Triggers RIG-I/TLR3/NLRP3-dependent Inflammasome Activation in Influenza A Virus Infected Cells
Source: PLoS Pathog. 2013 Apr 11;9(4):e1003256. doi: 10.1371/journal.ppat.1003256 (PMC3623797; doi:10.1371/journal.ppat.1003256)
Supplement: Table S4 — qRT-PCR primer sequences. (RTF) [file ppat.1003256.s010.rtf]

Table S4: qRT-PCR primer sequencesGenes	Primer Sequence 5′3′	m (°C)	SYBR Green Mix	
IAV M2	F:AAGACCAATCCTGTCACCTCTGA	60	QuantiTect	
 	R:CAAAGCGTCTACGCTGCAGTCC	 	 	
hβactin	F:AAGGAGAAGCTGTGCTAGGTCGC	54	QuantiTect	
 	R:AGACAGCACTGTGTTGGCGTACA	 	 	
hRIG-I	F:CTCTGCAGAAAGTGCAAAGC	60	Platinum	
 	R:GGCTTGGGATGTGGTCTACT	 	 	
hTLR3	F:GGTCCCAAGCCTTCAACGA	60	Platinum	
 	R:GGTGAAGGAGAGCTATCCACATTT	 		
hNLRP3	F:AAAGAGATGAGCCGAAGTGGG	60	Platinum	
 	R:TCAATGCTGTCTTCCTGGCA	 	 	
hRiplet	F:GTGGATCTTTCCATGGCTTC	60	QuantiTect	
 	R:AGTTCTCCCTGCATTTGTGC	 	 	
hIFN-β	:CATTACCTGAAGGCCAAGGA	60	Platinum	
 	R:CAATTGTCCAGTCCCAGAGG	 	 	
hIFNAR1	F:AGAAGTACATTTAGAAGCTG	48	QuantiTect	
 	R:AGTGCTGCTTTAACTTT	 	 	
hASC	F:agccaggcctgcactttat	60	Platinum	
 	R:ctggtactgctcatccgtca	 	 	
hcaspase1	F:CGACAAGGTCCTGAAGGAGA	60	Platinum	
 	R:CCCTTTCGGAATAACGGAGT	 	 	
fIL-1β	:TTCTTTGAGGCTGATGGTCC	58	QuantiTect	
 	R:ACACGAAATGGCTCAGACTC	 	 	
fIFN-β	:ACACTGCACTGGCAGAAGGAACAT	58	QuantiTect	
 	R:TATTGTCCAGGCACAGACGCTGAA	 	 	
h=Human, f=FerretQuantiTect: QuantiTect SYBR Green PCR Kit, QiagenPlatinum: Platinum SYBR Green qPCR SuperMix UDG, Invitrogen
