# Supplementary material for: Type I IFN Triggers RIG-I/TLR3/NLRP3-dependent Inflammasome Activation in Influenza A Virus Infected Cells
Source: PLoS Pathog. 2013 Apr 11;9(4):e1003256. doi: 10.1371/journal.ppat.1003256 (PMC3623797; doi:10.1371/journal.ppat.1003256)
Supplement: Table S5 — Target sequence of short interfering RNA (siRNA) (RTF) [file ppat.1003256.s011.rtf]

Table S5: Target sequence of short interfering RNA (siRNA)Gene	Pool#	Target Sequence	
IFNAR1	M-020209-00	AGAUAAGGCAAUAGUGAUA	
 	 	GUCAGAAUAUUACUAGUAC	
 	 	GAAUUUACCUUCUCCGCGU	
 	 	GCGAAAGUCUUCUUGAGAU	
IFN-β	M-019656-01	GCUAAUGUACUGCAUAUGA	
 	 	UGGCUAAUGUCUAUCAUCA	
 	 	CAACAAGUGUCUCCUCCAA	
 	 	GGAAUGAGACUAUUGUUGA	
RIG-I	M-012511-01	CAACCGAUAUCAUUUCUGA	
 	 	AGACAUGGGUAUAGAGUUA	
 	 	GCACAGAAGUGUAUAUUGG	
 	 	CAGAAGAUCUUGAGGAUAA	
TLR3	M-007745-00	GAAGCUAUGUUUGGAAUUA	
 	 	GAUCAUCGAUUUAGGAUUG	
 	 	GAAGAGGAAUGUUUAAUCU	
 	 	CAACAUAGCCAACAUAAAU	
NLRP3	M-017367-00	GGAUCAAACUACUCUGUGA	
 	 	GCAAAGGGCCAUGGACUAU	
 	 	UGCAAGAUCUCUCAGCAAA	
 	 	GAAGUGGGGUUCAGAUAAU	
Riplet	L-007087-00	UAGAGAAGAGCAUCACAGA	
 	 	GGACCUGGCCGACAAGUAC	
 	 	UGGAACAUCUUGUAGACAU	
 	 	CAUCCAACCUUUAACUUGA	
TRIM25	L-006585-00	CGGAACAGUUAGUGGAUUU	
 	 	CAACAAGAAUACACGGAAA	
 	 	GCGGAUGACUGCAAACAGA	
 	 	GGGAUGAGUUCGAGUUUCU	
Caspase 1	M-004401-03	GACUCAUUGAACAUAUGCA	
		AGACAUCCCACAAUGGGCU	
		GAAUAUGCCUGUUCCUGUG	
		CCGCAAGGUUCGAUUUUCA	
MAVS	MAVS.1	UAGUUGAUCUCGCGGACGA	
 	MAVS.2	CCGUUUGCUGAAGACAAGA	
