# Supplementary material for: Type I IFN Triggers RIG-I/TLR3/NLRP3-dependent Inflammasome Activation in Influenza A Virus Infected Cells
Source: PLoS Pathog. 2013 Apr 11;9(4):e1003256. doi: 10.1371/journal.ppat.1003256 (PMC3623797; doi:10.1371/journal.ppat.1003256)
Supplement: References S1 — Supplemental data references. (RTF) [file ppat.1003256.s012.rtf]

Supplemental Data References1. Meunier, I., and von Messling, V. (2011). NS1-mediated delay of type I interferon induction contributes to influenza A virulence in ferrets. J Gen Virol 92, 1635-1644.2. Pothlichet, J., Chignard, M., and Si-Tahar, M. (2008). Cutting edge: innate immune response triggered by influenza A virus is negatively regulated by SOCS1 and SOCS3 through a RIG-I/IFNAR1-dependent pathway. J Immunol 180, 2034-2038.3. Pothlichet, J., Burtey, A., Kubarenko, A.V., Caignard, G., Solhonne, B., Tangy, F., Ben-Ali, M., Quintana-Murci, L., Heinzmann, A., Chiche, J.D., et al. (2009). Study of human RIG-I polymorphisms identifies two variants with an opposite impact on the antiviral immune response. PLoS One 4, e758
